# Evaluation of Biological Activity of New 1,2,4-Triazole Derivatives Containing Propionic Acid Moiety

**DOI:** 10.3390/molecules28093808

**Published:** 2023-04-29

**Authors:** Renata Paprocka, Małgorzata Wiese-Szadkowska, Przemysław Kołodziej, Jolanta Kutkowska, Sara Balcerowska, Anna Bogucka-Kocka

**Affiliations:** 1 Department of Organic Chemistry, Faculty of Pharmacy, Collegium Medicum in Bydgoszcz, Nicolaus Copernicus University in Toruń, Jurasza Str. 2, 85-089 Bydgoszcz, Poland; 2 Department of Immunology, Faculty of Pharmacy, Collegium Medicum in Bydgoszcz, Nicolaus Copernicus University in Toruń, M. Curie-Sklodowska Str. 9, 85-094 Bydgoszcz, Poland; mwiese@cm.umk.pl (M.W.-S.); sara.balcerowska@onet.pl (S.B.); 3 Chair and Department of Biology and Genetics, Faculty of Pharmacy, Medical University in Lublin, Chodźki Str. 4A, 20-093 Lublin, Poland; przemyslaw.kolodziej@umlub.pl (P.K.); anna.bogucka-kocka@umlub.pl (A.B.-K.); 4 Department of Genetics and Microbiology, Institute of Biological Sciences, Maria Curie-Skłodowska University, Akademicka Str. 19, 20-033 Lublin, Poland; jolanta.kutkowska@mail.umcs.pl

**Keywords:** amidrazones, cytokines, TNF-α, anti-inflammatory, *Rhabditis* sp., propanoic acid, 1,2,4-triazole derivatives

## Abstract

To this day, the quest to find new drugs is still a challenge due to the growing demands of patients suffering from chronic inflammatory diseases and the need for the individualization of therapy. The aim of this research was to synthesize new 1,2,4-triazole derivatives containing propanoic acid moiety and to investigate their anti-inflammatory, antibacterial and anthelmintic activity. Compounds **3a**–**3g** were obtained in reactions of amidrazones **1a**–**1g** with succinic anhydride. Several analyses of proton and carbon nuclear magnetic resonance (^1^H NMR, ^13^C NMR, respectively), as well as high-resolution mass spectra (HRMS), confirmed the structures of 1,2,4-triazole derivatives **3a**–**3g**. Toxicity, antiproliferative activity and influence on cytokine release (TNF-α: Tumor Necrosis Factor-α, IL-6: Interleukin-6, IFN-γ: Interferon-γ, and IL-10: Interleukin-10) of the compounds **3a**–**3g** were evaluated in peripheral blood mononuclear cells culture. Moreover, mitogen-stimulated cell culture was used for biological activity tests. The antimicrobial and anthelmintic activity of derivatives **3a**–**3g** were studied against Gram-positive and Gram-negative bacterial strains and *Rhabditis* sp. culture. Despite the lack of toxicity, compounds **3a**–**3g** significantly reduced the level of TNF-α. Derivatives **3a**, **3c** and **3e** also decreased the release of IFN-γ. Taking all of the results into consideration, compounds **3a**, **3c** and **3e** show the most beneficial anti-inflammatory effects.

## 1. Introduction

Inflammatory symptoms, such as redness, pain, fever and swelling, have been known for centuries. They are caused by the action of many molecules, including prostaglandins, leukotrienes, cytokines and others, which are the key players in the pathomechanism of inflammation [1,2,3,4,5]. Leukotrienes and prostaglandins are biologically active metabolites derived from arachidonic acid. Cytokines are glycoproteins, and due to their biological functions, they are also called signaling proteins. All of these mediators work as chemoattractants for immunological cells. The migration of neutrophils, lymphocytes and other leucocytes can stop the process of inflammation because they eliminate the spreading of bacteria or viruses and start the process of healing the injured tissue [6,7]. However, high levels of these pro-inflammatory factors (e.g., IL-6—Interlukin-6, TNF-α—Tumor Necrosis Factor-α, IL-1β—Interleukin-1β, prostaglandins, leukotrienes) result in severe tissue damage, organ failure, and even death [8]. Moreover, chronic inflammation, in which a permanent increase in the concentration of pro-inflammatory cytokines is observed, could be involved in the development of many conditions, including Alzheimer’s disease, rheumatoid arthritis, cancer, type 2 diabetes and many others [4,9]. The commonly used non-steroidal anti-inflammatory drugs (NSAID) can indirectly block the production of prostaglandins by nonselective inhibition of the cyclooxygenase enzymes [2].

In our previous works, we used lipopolysaccharide (LPS)—stimulated peripheral blood mononuclear cells (PBMC) as an in vitro model of inflammatory processes. This model is commonly used to study the immunomodulatory properties of compounds [10,11,12,13,14,15]. The PBMC produce pro-inflammatory cytokines spontaneously after contact with bacteria and/or endotoxin. Interestingly, LPS applied in the same doses as in the presented experiment is used to mimic the model of sepsis [16]. LPS exposure activates nuclear factors, including (NF)-κB, and induces rapid release of IL-8 (Interleukin-8), IL-6, TNF-α and IL-10 (Interleukin-10) [17]. The other mitogen—phytohaemagglutinin (PHA)—was also used for testing the anti-inflammatory activities of compounds. PHA stimulates the proliferation of T cells [18], contrary to LPS, which is a potent stimulant of B cells, monocytes and macrophages [19].

**Figure 1:**
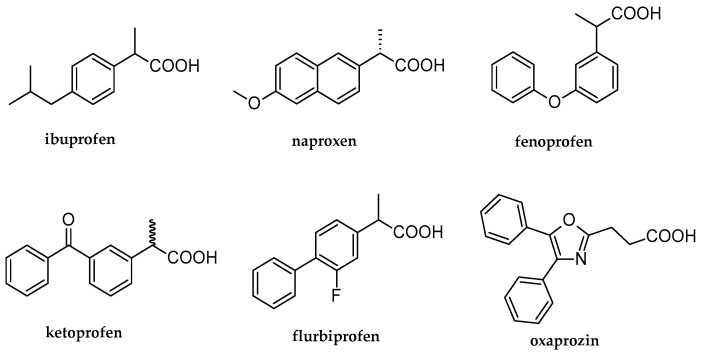
Selected structures of non-steroidal anti-inflammatory drugs (NSAID) containing propionic acid system.

The 1,2,4-triazole system is a well-known pharmacophore present in many drugs with different effects, e.g., antifungal (fluconazole), antiviral (ribavirin), sedative (alprazolam), anticancer (vorozole, anastrozole), antimigraine (rizatriptan), antihypertensive (trapidil) [20] and in other compounds with antitubercular and antibacterial activity [21,22]. Our previous studies showed that 1,2,4-triazole derivatives are an important group of anti-inflammatory compounds, particularly when linked to two aryl rings and an acrylic acid [23,24]. Since many NSAID such as ibuprofen, naproxen, fenoprofen, ketoprofen, flurbiprofen and oxaprozin contain a propionic acid moiety (Figure 1), we decided to obtain new derivatives containing this scaffold as a potential anti-inflammatory agent. Since amidrazone and 1,2,4-triazole derivatives can exhibit various biological activities [20,25,26,27], we also extended our research to antibacterial and anthelmintic activity.

The aim of this work was to obtain new 1,2,4-triazole derivatives substituted with propanoic acid moiety and to investigate their anti-inflammatory, antimicrobial and anthelmintic activity.

## 2. Results

### 2.1. The Synthesis of Compounds ***3a**–**3g***

**Scheme 1:**
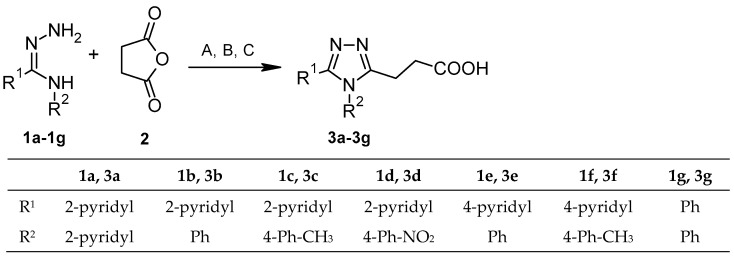
The general synthesis of compounds **3a**–**3g**. Method A: diethyl ether, ambient temp., 2 days; method B: boiling toluene 1–4 h; method C: boiling chloroform 1–1.5 h.

The reactions of amidrazones **1a**–**1g** [28] with succinic anhydride **2**, leading to 1,2,4-triazole derivatives **3a**–**3g**, could be carried out under different conditions, for instance, using catalyst HClO_4_-SiO_2_ without solvent [21]. In our earlier works [23,24], we mainly used diethyl ether as a solvent. This time we extended the research to toluene and chloroform (methods A–C, Scheme 1). For example, using diethyl ether as a solvent at room temperature (method A) obtained the best yield (85.76%) for compound **3b**, while the yields of the other reactions in this method were in the range of about 55.4–80.8% (Table S1, Supplementary Materials). However, carrying out the same reaction in boiling toluene (method B) allowed better yields to be obtained for **3e** and **3a** (85.71% and 69.36%, respectively) than for the same reactions carried out in diethyl ether. Due to the shorter reaction time, the use of boiling toluene as a solvent may be a good alternative to diethyl ether for the synthesis of 1,2,4-triazole derivatives. Boiling chloroform (method C) was less preferable as a solvent, probably due to its lower boiling point compared to toluene; only for compound **3e** was a yield above 65% obtained. However, other obtained yield values for chloroform were lower than those obtained in methods A and B (about 30–44.12%).

**Scheme 2:**
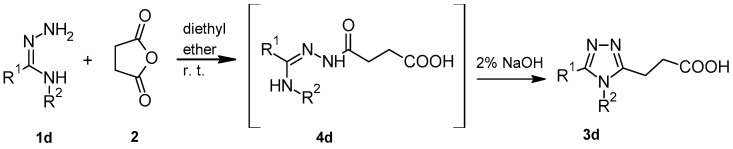
Synthesis of compound **3d** (method D).

Synthesis in diethyl ether (method A) failed to obtain compound **3d** containing an electron-withdrawing -NO_2_ group in the para position of the phenyl ring, the presence of which may hinder cyclization [22]. The intermediate compound **4d**, obtained in diethyl ether from amidrazone **1d**, was subjected to cyclization in an alkaline medium as previously described [29], obtaining compound **3d** (method D, Scheme 2).

The structures of derivatives **3a**–**3g** were confirmed by ^1^H NMR and ^13^C NMR spectra (Supplementary Materials, Figures S1–S14), high-resolution mass spectra (HRMS) (Supplementary Materials, Figures S15–S21) and elemental analyses, which are consistent with expected molecular structures. For example, in compound **3e**, the presence of a carboxyl group is confirmed by a broad signal at ca. 12.28 ppm in the ^1^H NMR spectrum and a signal at 173.60 ppm in the ^13^C NMR spectrum. The presence of methylene carbons –CH_2_- of derivative **3e** are characterized by two signals at 30.74 ppm and 20.72 ppm in the ^13^C NMR spectrum and a single peak at 2.73 ppm in the ^1^H NMR spectrum corresponding to the four hydrogen atoms attached to it.

In the ^1^H NMR spectra, a signal at ca. 8.55 ppm corresponds to two hydrogen atoms attached to aromatic carbon atoms attached to a nitrogen atom (in R^1^ substituent). The signals in the range of 7.62–7.26 ppm correspond to the remaining seven CH hydrogens attached to the pyridyl and phenyl rings. The ^13^C NMR spectrum is complemented by a group of signals in the range of ca. 156–122 ppm corresponding to aromatic carbon atoms. The value of the signal in the HRMS 295.1196 spectrum confirms the predicted molecular weight and composition of compound **3e**.

### 2.2. Biological Activity of Compounds ***3a**–**3g*** in PBMC

First, the toxicity of compounds **3a**–**3g** at the highest dose of 100 µg/mL was tested in a 24-h human PMBC culture. All derivatives (**3a**–**3g**) showed very low toxicity: the viable cell counts were in the range 94.71–96.72% and were comparable to the control culture with DMSO (94.19%) and culture with reference non-steroidal anti-inflammatory drug ibuprofen (IBU, 96.01%, Table S2, Supplementary Materials).

**Figure 2:**
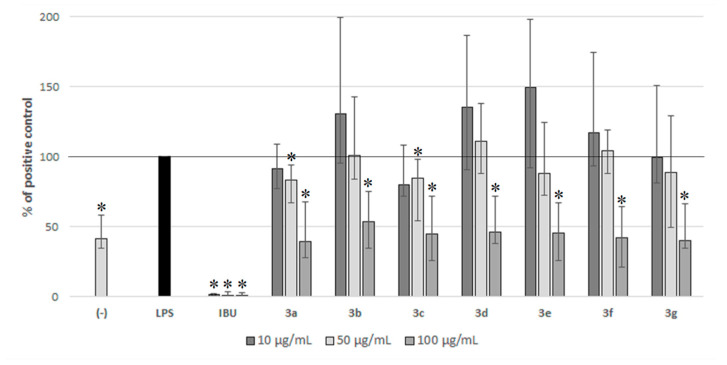
The effect of compounds **3a**–**3g** and ibuprofen (IBU) on the LPS (lipopolysaccharide)-induced production of TNF-α (Tumor Necrosis Factor-α) in 24 h PBMC (Peripheral Blood Mononuclear Cell) cultures; the results are shown as percentage of positive control—LPS alone; values are expressed as medians from four independent experiments and interquartile ranges [Q1, Q3]; * indicates a significant difference compared to the positive control at *p* < 0.05.

**Figure 3:**
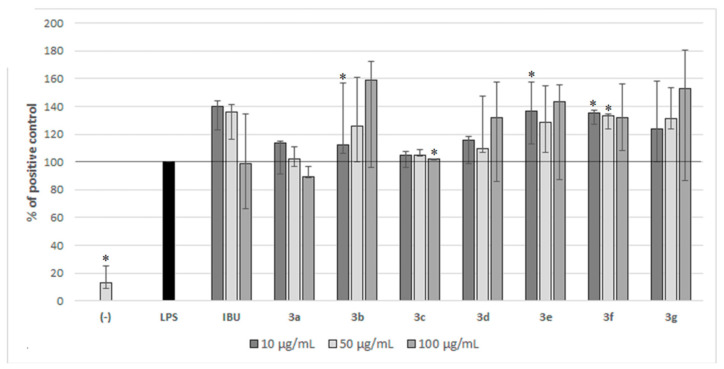
The effect of compounds **3a**–**3g** and ibuprofen (IBU) on the LPS (lipopolysaccharide)-induced production of IL-6 (Interleukin-6) in 24 h PBMC (Peripheral Blood Mononuclear Cell) cultures; the results are shown as percentage of positive control—LPS alone; values are expressed as medians from five independent experiments and interquartile ranges [Q1, Q3]; * indicates a significant difference compared to positive control at *p* < 0.05.

**Figure 4:**
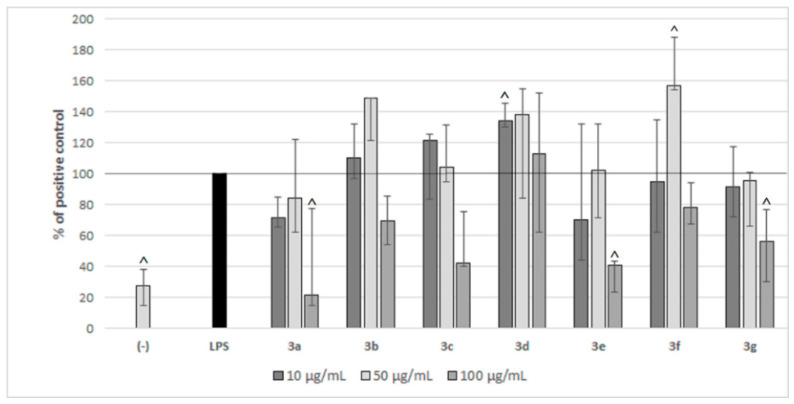
The effect of compounds **3a**–**3g** on the LPS (lipopolysaccharide)-induced production of IFN-γ (Interferon-γ) in 24 h PBMC (Peripheral Blood Mononuclear Cell) cultures; the results are shown as percentage of positive control—LPS alone; values are expressed as medians from three independent experiments and interquartile ranges [Q1, Q3]; ^ indicates a tendency compared to positive control at *p* = 0.0518.

**Figure 5:**
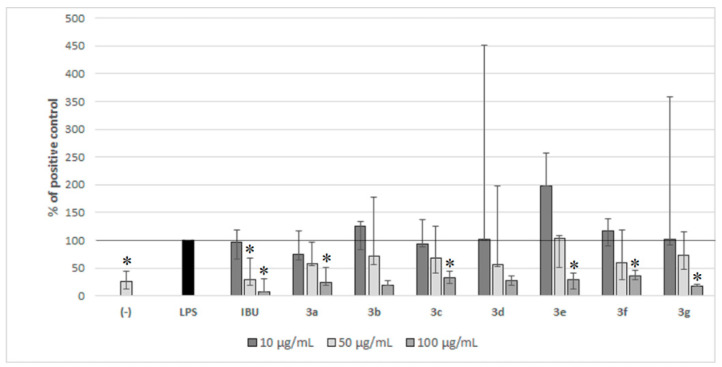
The effect of compounds **3a**–**3g** and ibuprofen (IBU) on the LPS (lipopolysaccharide)-induced production of IL-10 (Interleukin-10) in 24 h PBMC (Peripheral Blood Mononuclear Cell) cultures; the results are shown as percentage of positive control—LPS alone; values are expressed as medians from five independent experiments and interquartile ranges [Q1, Q3]; * indicates significant difference comparing to positive control at *p* < 0.05.

**Figure 6:**
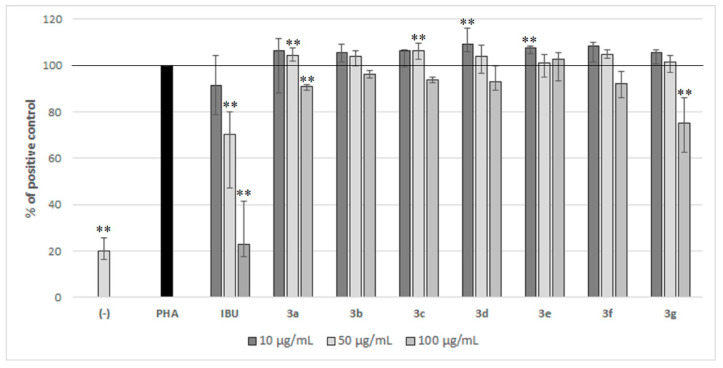
The effect of compounds **3a**–**3g** and ibuprofen (IBU) on the PHA (phytohaemagglutinin) -induced PBMC (Peripheral Blood Mononuclear Cell) proliferation in 72 h cell cultures; the results are shown as percentage of positive control—PHA alone; values are expressed as medians from six independent experiments and interquartile ranges [Q1, Q3]; ** indicates a significant difference compared to positive control at *p* < 0.01.

The influence of compounds **3a**–**3g** on cytokine release (TNF-α, IL-6, IFN-γ, and IL-10), as well as their antiproliferative activity, was evaluated in PBMC stimulated by LPS and PHA (respectively). The results of cytokine response and proliferation assays in PBMC cultures of compounds **3a**–**3g** are presented in Figure 2, Figure 3, Figure 4, Figure 5 and Figure 6.

All compounds **3a**–**3f** in the highest dose decreased production of TNF-α by about 44–60%, leading to a similar level of this cytokine as in control cells without stimulation (Figure 2). The strongest effects were exhibited by compounds **3a** and **3c**, which significantly inhibited TNF-α production in a medium dose of 50 µg/mL and, unlike compounds **3b** and **3d**–**3f**, did not increase the amount of TNF-a at the lowest dose.

Interestingly, only compounds **3a** and **3c** did not modulate LPS-induced IL-6 levels in PBMC culture (Figure 3). The rest of the tested compounds, including the reference ibuprofen, acted rather additively with LPS, increasing the level of this pro-inflammatory cytokine.

Compounds **3a**, **3c**, **3e** and **3g** inhibited the release of IFN-γ in the highest dose by about 44-79% (Figure 4). On the contrary, derivatives **3b**, **3d** and **3f** increased production of IFN-γ at the medium and low concentration

Ibuprofen significantly inhibited the production of IL-10 at medium and high doses by about 71% and 92%, respectively (Figure 5). At medium doses, all studied compounds were less effective than IBU, causing only about 27–45% inhibition of IL-10 release. Most of the studied compounds at the highest dose decreased the level of IL-10 by about 72–82%; only compounds **3e** and **3c** showed a slightly weaker effect (about a 65–68% decrease). Interestingly, LPS induced PBMC together with low doses of compounds **3b**–**3g** and IBU resulting in significantly higher levels of IL-10 than in the control cells without stimulation. The most favorable effect on the release of IL-10 was observed for compound **3e** (R^1^= 4-Pyr, R^2^ = Ph), which at the lowest dose increased the level of IL-10—this could be beneficial in relieving chronic inflammation.

IBU in the concentration of 100 µg/mL decreased the proliferation of PBMC to a level similar to the negative control (Figure 6). Studied compounds were less effective, only compounds **3g** and **3a** significantly inhibited the proliferation of PBMC in the highest dose by about 25% and 9% (respectively).

### 2.3. Anthelmintic Activity

**Figure 7:**
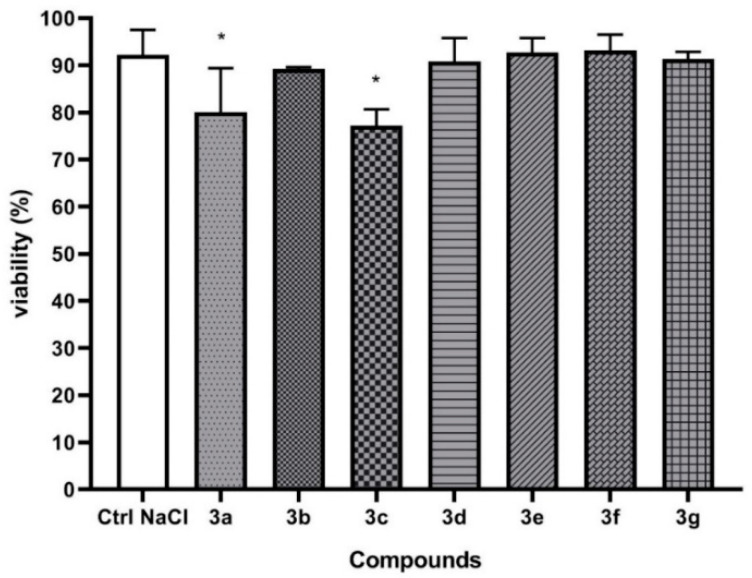
Nematodes of the genus *Rhabditis* sp. after 24 h of exposure to the highest concentration (11.1 mg/mL) of the tested compounds. * indicates significant difference comparing to control 0.6% NaCl (sodium chloride) at *p* < 0.05.

**Figure 8:**
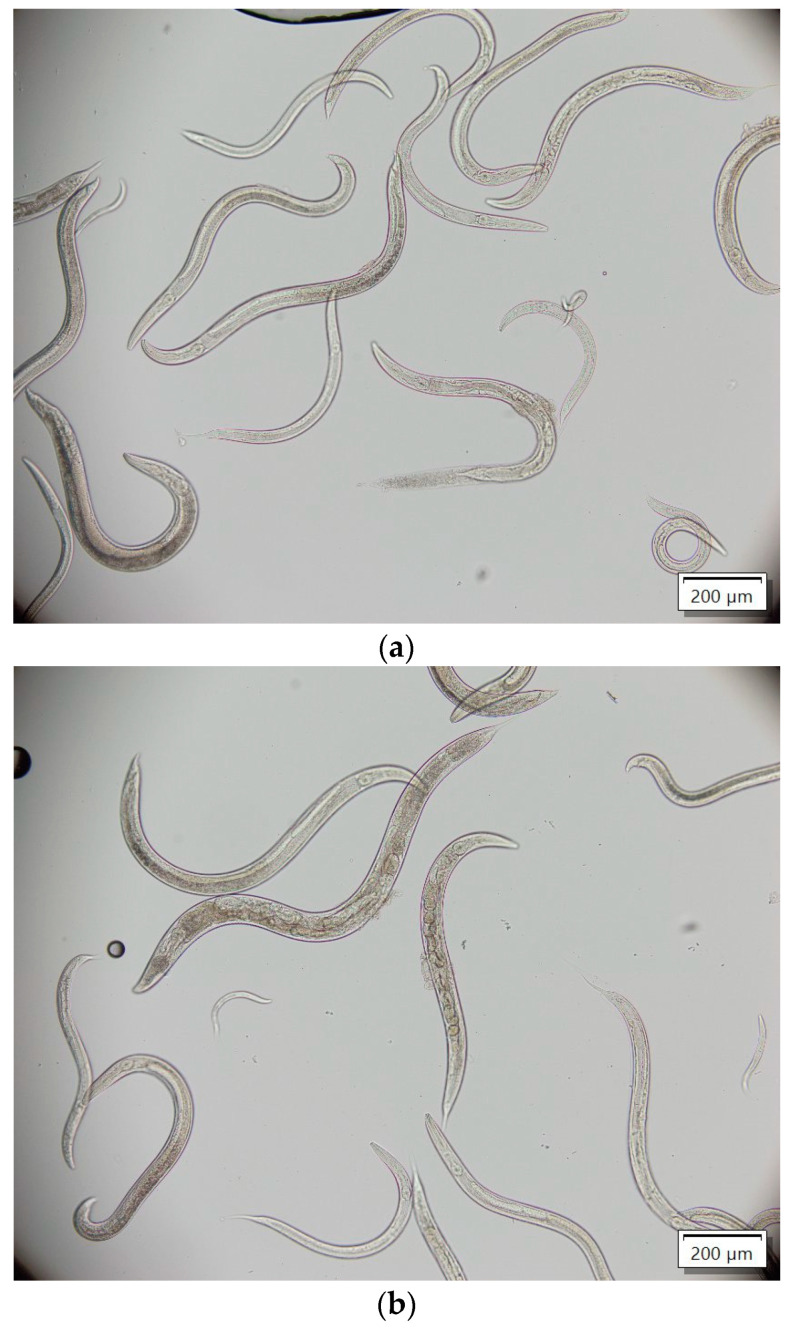
Example culture of *Rhabditis* sp.—alive and motile nematodes are visible. (**a**) control 0.6% NaCl (sodium chloride), (**b**) compound **3a**.

The studies of anthelmintic activity were carried out on a model of nematodes of the genus *Rhabditis* sp. The tested compounds **3a** and **3c** showed very low anthelmintic activity. The remaining compounds **3b** and **3d**–**3g** showed a lack of anthelmintic activity. Due to the high viability of the nematodes, the LC_50_ dose could not be determined (Figure 7 and Figure 8).

### 2.4. Antibacterial Activity

Tested compounds **3a**–**3g** showed low antimicrobial activity (MIC values ≥ 512 μg/mL) (Table S3, Supplementary Materials) and were less effective than ciprofloxacin (CIP) and amphotericin B (AmB) used as antibacterial and antifungal reference drugs. However, five compounds exhibited anti-mycobacterial activity: **3a** and **3e** at a concentration of 128 μg/mL and **3b**, **3c** and **3f** at a concentration of 256 μg/mL. Only the derivative **3g** inhibited the growth of *E. coli* and *S. aureus* at a concentration of 256 μg/mL.

## 3. Discussion

The toxicity of all obtained compounds **3a**–**3g** was tested and was comparable with the control culture (PBMC without additions). This confirms that we work with non-toxic compounds (viability of cells around 90%).

The promising compounds could be **3a** containing two 2-pyridine (2-Pyr) substituents, **3c** (R^1^ = 2-Pyr, R^2^ = 4-Ph-CH_3_) and **3e** (R^1^ = 4-Pyr, R^2^ = Ph) since these compounds inhibit the TNF-α and IFN-γ release. Additionally, derivative **3e** showed favorable activity in inducting the IL-10 production. It should be noted that these promising compounds immunomodulate innate and adaptive immunity alike since both levels of TNF-α and INF-γ were altered. This is certainly related to the activity of lymphocytes and monocytes [30]. The anti-inflammatory and immunomodulatory activity observed in compounds **3a**–**3f** is not related to the inhibition of the proliferation of the test cells because the level of this inhibition is very low (Figure 6).

In previous in vitro studies of acetylsalicylic acid and ibuprofen, they inhibited the production of IL-17 and TNF-α when used at a concentration range of 25–100 µg/mL. However, no significant effect on IFN-γ release was observed [31]. It may suggest that compounds **3a**, **3c** and **3e** could possess an additional beneficial component of the mechanism (IFN-γ inhibition) compared to the anti-inflammatory drugs mentioned above.

Comparing the biological activity of the obtained derivatives **3a**–**3g**, some crucial relationships could be observed between the different R^1^ and R^2^ substitutions in the 1,2,4-triazole ring, kind of aliphatic acid moiety and the biological effects of those compounds.

1. The presence of 2-pyridyl substituents in R^1^ position increased TNF-α inhibitory activity of compounds **3a** and **3c**;
2. The presence of two 2-pyridyne substituents (**3a**), 4-pyridyl substituent in R^1^, and phenyl ring in R^2^ (**3e**) or two phenyl rings in R^1^ and R^2^ (**3g**) improved the ability to decrease IFN-γ levels. Observations for the last two types of substitutions are consistent with the previously described observations for methacrylic acid derivatives [25];
3. The presence of 4-pyridyne substituent in R^1^ and phenyl ring in R^2^ positions in **3e** derivative had beneficial influence on the level of IL-10 cytokine;
4. The presence of two phenyl or two 2-pyridyl substituents on R^1^ and R^2^ positions seemed to increase the antiproliferative activity of **3g** and **3a** derivatives;
5. The presence of two 2-pyridyne substituents in R^1^ and R^2^ or 2-pyridyne substituent in R^1^ and 4-methylphenyl substituent in R^2^ seemed to increase the anthelmintic potential of compounds **3a** and **3c**;
6. The presence of two 2-pyridyl substituents or 4-pyridyl and phenyl substituents in R^1^ and R^2^ positions increased the antituberculosis activity;
7. The presence of the propionic acid group reduced the antibacterial activity of compounds **3a**–**3g** compared to analogous 1,2,4-triazole derivatives substituted with methacrylic acid [32].

Comparing the antibacterial and anthelmintic activity of compounds **3a**–**3g** with the activity of the previously described 1,2,4-triazole derivatives with similar structures containing the propenoic [33] or methacrylic acid system [32], it can be concluded that the lower activity of the obtained compounds is probably related to the lack of a double bond in the acid chain in their molecules. For example, compounds **3c** and **3e** were less effective against nematodes than their methacrylic acid analogues. Also, the previously described 1,2,4-triazole derivatives showed a stronger inhibitory effect on PBMC proliferation and TNF-α release [23,24], which emphasizes the beneficial effect of the aliphatic acid substituent containing a double bond and a branched chain. However, the strongest anti-inflammatory, anti-mycobacterial and anthelmintic activities among derivatives **3a**–**3g** were observed for compounds **3a** and **3c**, which confirmed that they have the highest biological potential among the studied compounds.

However, there are some limitations to this immunomodulatory study. The PBMC culture is taken from the vein blood of healthy donors, but there are some individual factors that could influence the presented results. However, this is a useful test system for the investigation of immune modulatory effects of newly synthesized chemical compounds. For future investigation, we plan to additionally use human cell lines. Furthermore, we check the limited number of pro- and anti-inflammatory factors, such as IL-6, TNF-α, IFN-γ and IL-10. There are more factors that affect the inflammatory system, and this may be a new direction for future study (for example, prostaglandins and leukotrienes or cytokines such as IL-17).

## 4. Materials and Methods

### 4.1. General Information

^1^H NMR and ^13^C NMR spectra: the Bruker Avance 300, 400 and 700 apparatus (TMS as an internal standard). Melting points: MEL-Temp apparatus (Electrothermal, Stone, UK). Elemental analyses: CHN Vario MACRO analyzer (Elementar Analysensysteme GmbH, Langenselbold, Germany). HRMS (high-resolution mass spectrometry): Synapt G2 Si mass spectrometer (Waters). The retention factors: reverse-faced plates (nano-silica gel RP-18W on alu. foil with fluorescent indicator (Merck, Darmstadt, Germany) and a methanol-water (1:1) mixture. All reactions were controlled by TLC chromatography. Received yields and melting points using particular methods of synthesis are presented in Table S1 in Supplementary Materials. The ^1^H NMR and ^13^C NMR spectra (Figures S1–S14) and their HRMS spectra (Figures S15–S21) are present in Supplementary Materials.

### 4.2. General Procedures of Synthesis

#### 4.2.1. Method A (Synthesis of Compounds **3a**–**3c** and **3e**–**3g**)

In total, 1.2 mmol of amidrazones **1a**–**1c** and **1e**–**1g** [28] and 1.2 mmol (0.12 g) of succinic anhydride **2** were dissolved in 30 mL of anhydrous diethyl ether, stirred for 2 h, then left for 2 days in ambient temperature. Obtained crude compounds **3a**–**3c** and **3e**–**3g** were filtered and washed with anhydrous diethyl ether and products were crystallized from 1:1 methanol/water mixture.

#### 4.2.2. Method B (Synthesis of Compounds **3a**–**3g**)

In total, 1.2 mmol of amidrazones **1a**–**1g** [28] and 1.2 mmol (0.12 g) of succinic anhydride **2** were dissolved in 30 mL of anhydrous toluene. The mixtures were heated under reflux for 1–1.5 h, then filtered out. Crude compounds **3a**–**3g** obtained by evaporation of toluene were washed with anhydrous diethyl ether. Received products **3a**–**3g** were crystallized (**3a**, **3c** and **3g**: 1:1 ethanol/water mixture, **3b**, **3d** and **3e**: water).

#### 4.2.3. Method C (Synthesis of Compounds **3a**–**3c** and **3e**)

In total, 1.2 mmol of amidrazones **1a**–**1c** and **1e** [28] and 1.2 mmol (0.12 g) of succinic anhydride **2** were dissolved in 30 mL of anhydrous chloroform. The mixtures were heated under reflux for 1–4 h, then filtered out. Crude compounds obtained by partial evaporation of chloroform were filtered and dried. Received products **3a**–**3c** were crystallized (**3a**: ethanol, **3b**–**3c**: water).

#### 4.2.4. Method D (Compound **3d**)

In total, 1.2 mmol of amidrazone **1d** [28] and 1.2 mmol (0.12 g) of succinic anhydride **2** were dissolved in 30 mL of anhydrous diethyl ether, stirred for 2, then left for 2 days in ambient temperature. Obtained intermediate **4d** was filtered and washed with anhydrous diethyl ether and dried. Then the cyclization procedure was undertaken by heating in 2% aqueous sodium hydroxide solution for 2 h. After filtration and cooling obtained solution and neutralization with 2% aqueous hydrochloric acid solution. Obtained compound **3d** was crystalized by isopropanol.

3-(4,5-di(pyridin-2-yl)-4*H*-1,2,4-triazol-3-yl)propanoic acid (**3a**): yield 55.39%, m.p. 197–199 °C. ^1^H NMR (700 MHz, DMSO-d_6_, δ ppm): 12.25 (sb, 1H, COOH), 8.56 (d, 1H, *J* = 4.0 Hz), 8.23, (d, 1H, *J* = 4.0 Hz), 8.07–7.89 (m, 3H), 7.58–7.50 (m, 2H), 7.38–7.33 (m, 1H), 2.85–2.67 (m, 4H). ^13^C NMR (100 MHz, DMSO-d_6_, δ ppm): 173.6, 155.3, 152.8, 149.7, 149.3, 148.9, 146.8, 139.6, 137.8, 125.0, 124.8, 123.6, 122.8, 30.8, 20.9. HR-MS *m/z:* 296.1150 [M^+^ + 1] (calculated for C_15_H_14_N_5_O_2_: 296.1147). R_f_ = 0.53. Elem. Anal. for C_15_H_13_N_5_O_2_ calculated: C, 61.01; H, 4.44; N, 23.72%; obtained C, 61.18; H, 4.67; N, 23.63%.
3-(4-phenyl-5-(pyridin-2-yl)-4*H*-1,2,4-triazol-3-yl)propanoic acid (**3b**): yield 85.76%, m.p. 178–180 °C. ^1^H NMR (400 MHz, DMSO-d_6_, δ ppm): 12.26 (sb, 1H, COOH), 8.30–8.28, (m, 1H), 7.97–7.87 (m, 2H), 7.52–7.47 (m, 3H), 7.37–7.32 (m, 3H), 2.72 (s, 4H). ^13^C NMR (100 MHz, DMSO-d_6_, δ ppm): 173.6, 155.7, 152.9, 149.4, 147.2, 137.6, 135.5, 129.8 (2×), 129.5, 127.8 (2x), 124.6, 124.1, 30.8, 20.8. HR-MS *m/z:* 29.1197 [M^+^ + 1] (calculated for C_16_H_15_N_4_O_2_: 295.1195). R_f_ = 0.41. Elem. anal. for C_16_H_14_N_4_O_2_ * H_2_O calculated: C, 61.54; H, 5.13; N, 17.95%; obtained C, 61.89; H, 5.08; N, 17.72%.
3-(5-(pyridin-2-yl)-4-*p*-tolyl-4*H*-1,2,4-triazol-3-yl)propanoic acid (**3c**): yield 73.53%, m.p. 157–160 °C. ^1^H NMR (700 MHz, DMSO-d_6_, δ ppm): 12.24 (sb, 1H, COOH), 8.32 (d, 1H, *J* = 4.2 Hz), 7.92 (d, 1H, *J* = 7.7 Hz), 7.89–7.86 (m, 1H), 7.35–7.32 (m, 1H), 7.28 (d, 2H, *J* = 8.4 Hz), 7.22 (d, 2H, *J* = 8.4 Hz), 2.72–2.67 (m, 4H), 2.36 (s, 3H). ^13^C NMR (100 MHz, DMSO-d_6_, δ ppm): 173.6, 155.7, 153.0, 149.5, 147.3, 139.0, 137.5, 132.9, 130.3 (2×), 127.6 (2×), 124.6, 124.2, 30.8, 21.2, 20.8. HR-MS *m/z:* 309.1357 [M^+^ + 1] (calculated for C_17_H_17_N_4_O_2_: 309.1352). R_f_ = 0.32. Elem. Anal. for C_17_H_16_N_4_O_2_ calculated: C, 66.22; H, 5.23; N, 18.17%; obtained C, 66.56; H, 5.25; N, 18.17%.
3-(4-(4-nitrophenyl)-5-(pyridin-2-yl)-4*H*-1,2,4-triazol-3-yl)propanoic acid (**3d**): yield 32.83%, m.p. 189–191 °C. ^1^H NMR (400 MHz, DMSO-d_6_, δ ppm): 12.29 (sb, 1H, COOH), 8.36 (d, 2H, *J* = 9.2 Hz), 8.26 (d, 1H, *J* = 4.4 Hz), 8.09 (d, 1H, *J* = 8.0 Hz), 7.93 (td, 1H, *J*_1_ = 8 Hz, *J*_2_ = 1.6 Hz), 7.75–7.69 (m, 2H), 7.39–7.34 (m, 1H), 2.75 (s, 4H). ^13^C NMR (100 MHz, DMSO-d_6_, δ ppm): 173.3. 155.2, 152.2, 148.9, 147.5, 146.4, 141.1, 137.5, 129.2 (2x), 124.7 (2×), 124.5, 123.4, 30.6, 20.5. HR-MS *m/z:* 340.1049 [M^+^ + 1] (calculated for C_16_H_14_N_5_O_4_: 340.1046). ESI—MS *m/z:* (M + 1): 340. R_f_ = 0.38. Elem. Anal. for C_16_H_13_N_5_O_4_ calculated: C, 56.64; H, 3.86; N, 20.64%; obtained C, 56.56; H, 3.92; N, 20.51%.
3-(4-phenyl-5-(pyridin-4-yl)-4*H*-1,2,4-triazol-3-yl)propanoic acid (**3e**): yield 80.82%, m.p. 235–237 °C. ^1^H NMR (700 MHz, DMSO-d_6_, δ ppm): 12.28 (sb, 1H, COOH), 8.54–8.55 (m, 2H), 7.59–7.63 (m, 3H), 7.47–7.52 (m, 2H), 7.25–7.29 (m, 2H), 2.73 (s, 4H). ^13^C NMR (100 MHz, DMSO-d_6_, δ ppm): 173.6, 156.1, 151.5, 150.5 (2×), 134.8, 134.3, 130.7 (2×), 130.7, 128.0 (2×), 122.1 (2×), 30.7, 20.7. HR-MS *m/z:* 295.1196 [M^+^ + 1] (calculated for C_16_H_15_N_4_O_2_: 295.1195). R_f_ = 0.38. Elem. Anal. for C_16_H_14_N_4_O_2_ calculated: C, 61.54; H, 5.13; N, 17.95%; obtained C, 61.58; H, 5.12; N, 17.96%.
3-(5-(pyridin-4-yl)-4-*p*-tolyl-4*H*-1,2,4-triazol-3-yl)propanoic acid (**3f**): yield 64.30%, m.p. 208–211 °C. ^1^H NMR (300 MHz, DMSO-d_6_, δ ppm): 12.26 (sb, 1H, COOH), 8.56–8.52, (m, 2H), 7.41–7.24 (m, 6H), 2.70 (s, 4H), 2.39 (s, 3H). ^13^C NMR (100 MHz, DMSO-d_6_, δ ppm): 173.6, 156.2, 151.5, 150.5 (2×), 140.5, 134.9, 131.7, 131.2 (2×), 127.8 (2×), 122.1 (2×), 30.8, 21.2, 20.7. R_f_ = 0.28. HR-MS *m/z:* 309.1355 [M^+^ + 1] (calculated for C_17_H_17_N_4_O_2_: 309.1352). Elem. Anal. for C_17_H_16_N_4_O_2_ * ½ H_2_O calculated: C, 64.40; H, 5.36; N, 17.68%; obtained C, 64.58; H, 5.47; N, 17.36%.
3-(4,5-diphenyl-4*H*-1,2,4-triazol-3-yl)propanoic acid (**3g**): yield 76.47%, m.p. 251–254 °C. ^1^H NMR (400 MHz, DMSO-d_6_, δ ppm): 12.23 (sb, 1H, COOH), 7.57–7.52 (m, 3H), 7.43–7.29 (m, 7H), 2.70 (s, 4H). ^13^C NMR (100 MHz, DMSO-d_6_, δ ppm): 173.6, 155.0, 153.6, 134.8, 130.5 (2×), 130.2, 129.9, 129.0 (2×), 128.4 (2×), 128.1 (2×), 127.5, 30.8, 20.8. R_f_ = 0.31. HR-MS *m/z:* 294.1240 [M^+^ + 1] (calculated for C_17_H_16_N_3_O_2_: 294.1243). Elem. Anal. for C_17_H_15_N_3_O_2_ calculated: C, 69.61; H, 5.15; N, 14.33%; obtained C, 69.69; H, 5.26; N, 14.33%.

### 4.3. Biological Activity Evaluation for Compounds ***3a**–**3g***

#### 4.3.1. Cell Culture Preparation and Toxicity of Compounds **3a**–**3g** towards PBMC

Biological activity was evaluated on fresh peripheral blood taken from healthy donors. Informed consent was obtained from donors at the Occupational Medicine Clinic located in Dr. Antoni Jurasz University Hospital in Bydgoszcz, Poland. Immediately after the blood was taken, the cell isolation was performed by using the density gradient centrifugation (Lymphosept, BioWest, Nuaillé, France) as previously described [23]. After isolation, PBMC were used to conduct experiments which assessed toxicity of compounds and their impact on proliferation and cytokine production. The preparation of cell culture was described previously [23]. PBMC were stimulated with all tested compounds in highest doses (100 µg/mL). Additionally, we used ibuprofen as a reference drug in this study. For checking the number of viable cells, the Annexin V Apoptosis Detection Kit I (Becton Dickinson, Franklin Lakes, NJ, USA) was used. The procedure of staining was described previously [23].

#### 4.3.2. The Influence of Compounds **3a**–**3g** on Cytokines Production in PBMC

Cytokines (TNF-α, IL-6, IL-10 and IFN-γ) supernatant levels were measured as previously described [23] using the commercially available ELISA kits (DuoSet, BD Bioscience, San Diego, CA, USA). Tests were conducted according to the manufacturer’s instructions. The supernatants from cell culture were analyzed with iEMS Reader MF (Labsystems, Vantaa, Finland) and date were calculated by Ascent Software AscSW26 (London, UK).

#### 4.3.3. The Influence of Compounds **3a**–**3g** on PBMC Proliferation

Lymphocyte proliferation was evaluated by flow cytometry using BD Horizon^TM^ Violet Proliferation Dye 450 (VPD450, BD Pharmingen, San Diego, CA, USA). Violet Proliferation Dye 450 is a tool for evaluation of cell division. VPD450-stained cells were cultured for 72 h with the mitogen, phytohemagglutinin (PHA, Sigma-Aldrich, 1 μg/mL, positive control), and/or increasing concentrations of compounds (10, 50 and 100 μg/mL). The procedure of staining with VPD450 was described previously [23]. The cells were analyzed by flow cytometry (FACSCanto II flow cytometer, Becton Dickinson, Franklin Lakes, NJ, USA). Twenty thousand events were collected and analyzed with FlowJo software (v 7.6.1, Tree Star, Ashland, OR, USA). 

### 4.4. Anthelmintic Activity of Compounds **3a**–**3g**

In vitro experiments on anthelmintic activity were carried out using a nematode model of the genus *Rhabditis* sp. The tests were conducted in accordance with the patented procedure patent No. PL232918. The compounds **3a**–**3g** were tested in five concentrations: 0.2; 1.1; 3.3; 5.5; 11.1 mg/mL. The exact methodology of testing anthelmintic activity was described in our earlier publication [23].

### 4.5. Antibacterial Activity of Compounds **3a**–**3g**

The antibacterial activity of compounds **3a**–**3g** was tested by the broth microdilution method against bacteria *Staphylococcus aureus* ATCC 25923, *Escherichia coli* ATCC 25922, *Yersinia enterocolitica* O3 and *Mycobacterium smegmatis* and fungal strain *Candida albicans* ATCC 90028. Compounds **3a**–**3g** were dissolved, serially diluted to concentrations from 512 to 0.25 μg/mL and then inoculated with bacterial cultures as described previously [34].

In addition, the sensitivity of the tested bacterial strains to ciprofloxacin (quinolone antibiotic) and the yeast strain to amphotericin B was determined. After 18 h of incubation at 37 °C, the minimum inhibitory concentration (MIC) of the compounds was defined. The experiments were carried out three times.

### 4.6. Data Analysis

The statistical analyses were performed using Statistica 13.3. software (StatSoft, Cracow, Poland). Shapiro-Wilka test and Mann-Whitney U test were calculated. All *p*-values represented the nonparametric Mann–Whitney U test. The *p* value was set at 0.05.

## 5. Conclusions

Seven new 1,2,4-triazole derivatives **3a**–**3g** were obtained through the reaction of amidrazones **1a**–**1g** with succinic anhydride. It was shown that the use of boiling toluene is a good alternative for the synthesis of this type of cyclic compound in relation to the classical reaction carried out in diethyl ether. All obtained derivatives **3a**–**3g** showed anti-inflammatory and immunomodulatory activity. The strongest effect was shown by derivatives **3a**, **3c** and **3e**, which decreased levels of TNF-α and IFN-γ in mitogen-stimulated PBMC cultures. Compared to the previously described 1,2,4-triazole derivatives containing the methacrylic acid system, lower antibacterial, antiproliferative and anthelmintic activity was observed for compounds **3a**–**3g**. This decrease in biological activity could be connected to the lack of a double bond and chain branching in the aliphatic side chain of the acid. The mechanisms of observed effects require further investigation.

## Data Availability

Data available from the authors.

## Supplementary Materials

The following supporting information can be downloaded at: https://www.mdpi.com/article/10.3390/molecules28093808/s1. Table S1. A summary of the yield and melting point of compounds **3a**–**3g** obtained under different reaction conditions (methods A–D). Figure S1. ^1^H NMR spectrum of compound **3a** (in DMSO-d_6_). Figure S2. ^1^H NMR spectrum of compound **3b** (in DMSO-d_6_). Figure S3. ^1^H NMR spectrum of compound **3c** (in DMSO-d_6_). Figure S4. ^1^H NMR spectrum of compound **3d** (in DMSO-d_6_). Figure S5. ^1^H NMR spectrum of compound **3e** (in DMSO-d_6_). Figure S6. ^1^H NMR spectrum of compound **3f** (in DMSO-d_6_). Figure S7. ^1^H NMR spectrum of compound **3g** (in DMSO-d_6_). Figure S8. ^13^C NMR spectrum of compound **3a** (in DMSO-d_6_). Figure S9. ^13^C NMR spectrum of compound **3b** (in DMSO-d_6_). Figure S10. ^13^C NMR spectrum of compound **3c** (in DMSO-d_6_). Figure S11. ^13^C NMR spectrum of compound **3d** (in DMSO-d_6_). Figure S12. ^13^C NMR spectrum of compound **3e** (in DMSO-d_6_). Figure S13. ^13^C NMR spectrum of compound **3f** (in DMSO-d_6_). Figure S14. ^13^C NMR spectrum of compound **3g** (in DMSO-d_6_). Figure S15. HRMS spectrum of compound **3a**. Figure S16. HRMS spectrum of compound **3b**. Figure S17. HRMS spectrum of compound **3c**. Figure S18. HRMS spectrum of compound **3d**. Figure S19. HRMS spectrum of compound **3e**. Figure S20. HRMS spectrum of compound **3f**. Figure S21. HRMS spectrum of compound **3g**. Table S2. The results of cell apoptosis assay of compounds **3a**–**3g** and ibuprofen at a concentration of 100 µg/mL in PBMC 24 h cultured measured by flow cytometry. Table S3. Minimal inhibitory concentrations of compounds **3a**–**3g**, ciprofloxacin (CIP) and amphotericin B (AmB).

## Sample Availability

Samples of the compounds **3a**–**3g** are available from the authors.
