# Supplementary material for: Evaluation of Biological Activity of New 1,2,4-Triazole Derivatives Containing Propionic Acid Moiety
Source: Molecules. 2023 Apr 29;28(9):3808. doi: 10.3390/molecules28093808 (PMC10180335; doi:10.3390/molecules28093808)
Supplement: Supplementary file 1 [file molecules-28-03808-s001.zip › molecules-2348503-supplementary.pdf]

# Evaluation of biological activity of new 1,2,4-triazole derivatives containing propionic acid moiety

**Renata Paprocka [1,*,†], Małgorzata Wiese-Szadkowska [2,†], Przemysław Kołodziej [3], Jolanta Kutkowska [4], Sara Balcerowska [2], Anna Bogucka-Kocka [3]**

[1] Department of Organic Chemistry, Faculty of Pharmacy, Collegium Medicum in Bydgoszcz,
Nicolaus Copernicus University in Toruń, Jurasza Str. 2, 85-089 Bydgoszcz, Poland;

[2] Department of Immunology, Faculty of Pharmacy, Collegium Medicum in Bydgoszcz, Nicolaus Copernicus University in Toruń, M. Curie-Sklodowska Str. 9, 85-094 Bydgoszcz, Poland

[3] Chair and Department of Biology and Genetics, Faculty of Pharmacy, Medical University in Lublin,
Chodźki Str. 4A, 20-093 Lublin, Poland

[4] Department of Genetics and Microbiology, Institute of Biological Sciences, Maria Curie-Skłodowska University, Akademicka Str. 19, 20-033 Lublin, Poland

* Correspondence: renata.bursa@cm.umk.pl (R.P)
† These authors contributed equally to this work.

Table S1. A summary of the yield and melting point of compounds **3a-3g** obtained under different reaction conditions (methods A-D)

| compound | method | solvent | temp. | time | yield | melting point |
|---|---|---|---|---|---|---|
| **3a** | A | diethyl ether | ambient | 2 days | 55.39% | 197-199 °C |
| **3a** | B | toluene | boiling | 4 h | 69.36% | 199-202 °C |
| **3a** | C | chloroform | boiling | 1 h | 29.90% | 198-200 °C |
| **3b** | A | diethyl ether | ambient | 2 days | 85.76% | 178-180 °C |
| **3b** | B | toluene | boiling | 1 h | 52.94% | 181-183 °C |
| **3b** | C | chloroform | boiling | 1.5 h | 44.12% | 183-185 °C |
| **3c** | A | diethyl ether | ambient | 2 days | 73.53% | 157-160 °C |
| **3c** | B | toluene | boiling | 1 h | 31.58% | 157-159 °C |
| **3c** | C | chloroform | boiling | 1 h | 35.29% | 158-160 °C |
| **3d** | B | toluene | boiling | 2.5 h | 32.83% | 186-189 °C |
| **3d** | D | alkaline cyclization* | boiling | 2 h | 31.88% | 189-191 °C |
| **3e** | A | diethyl ether | ambient | 2 days | 80.82% | 235-237 °C |
| **3e** | B | toluene | boiling | 1 h | 85.71% | 237-240 °C |
| **3e** | C | chloroform | boiling | 1.5 h | 66.47% | 237-239 °C |
| **3f** | A | diethyl ether | ambient | 2 days | 64.30% | 208-211 °C |
| **3f** | B | toluene | boiling | 1 h | 53.79% | 208-211 °C |
| **3g** | A | diethyl ether | ambient | 2 days | 76.47% | 251-254 °C |
| **3g** | B | toluene | boiling | 1.5 h | 74.93% | 250-252 °C |

*alkaline cyclization of intermediate obtained in diethyl ether

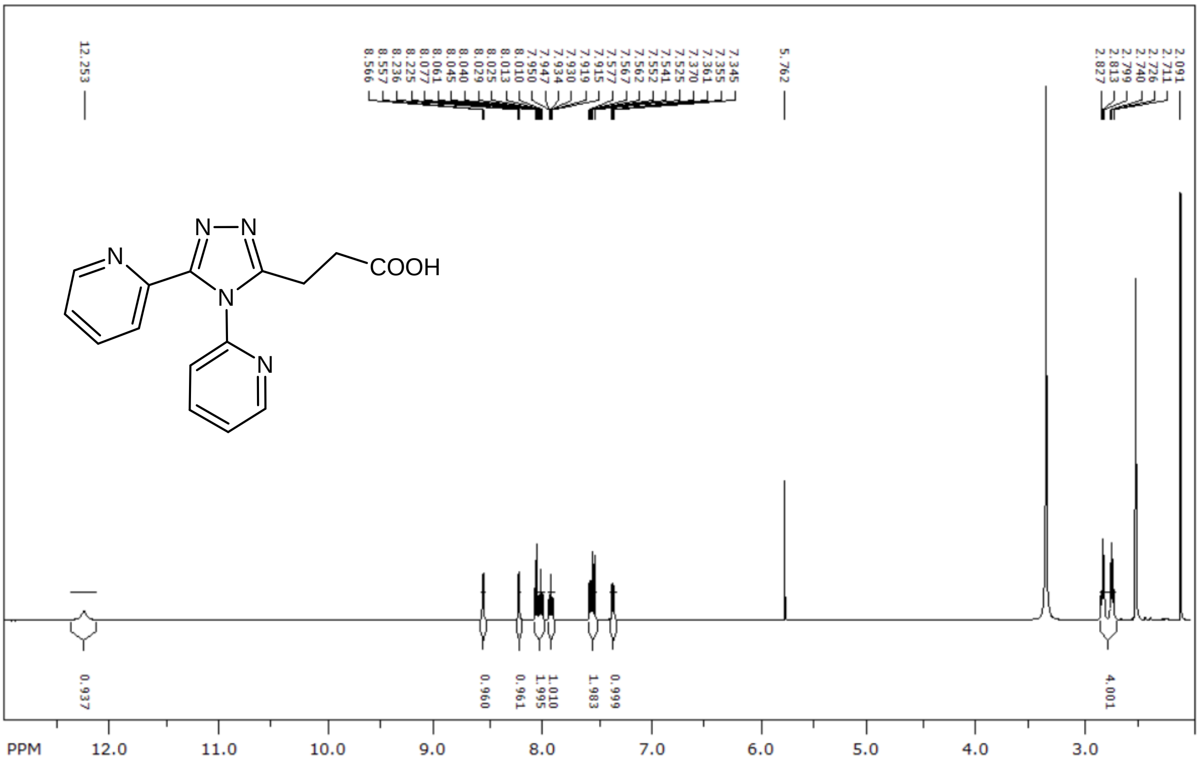


Figure S1. $^{1}$H NMR spectrum of compound **3a** (in DMSO-$d_6$)

(5.762 ppm – impurity of dichloromethane formed in the NMR study, 2.091 – impurity of acetone in the NMR study)

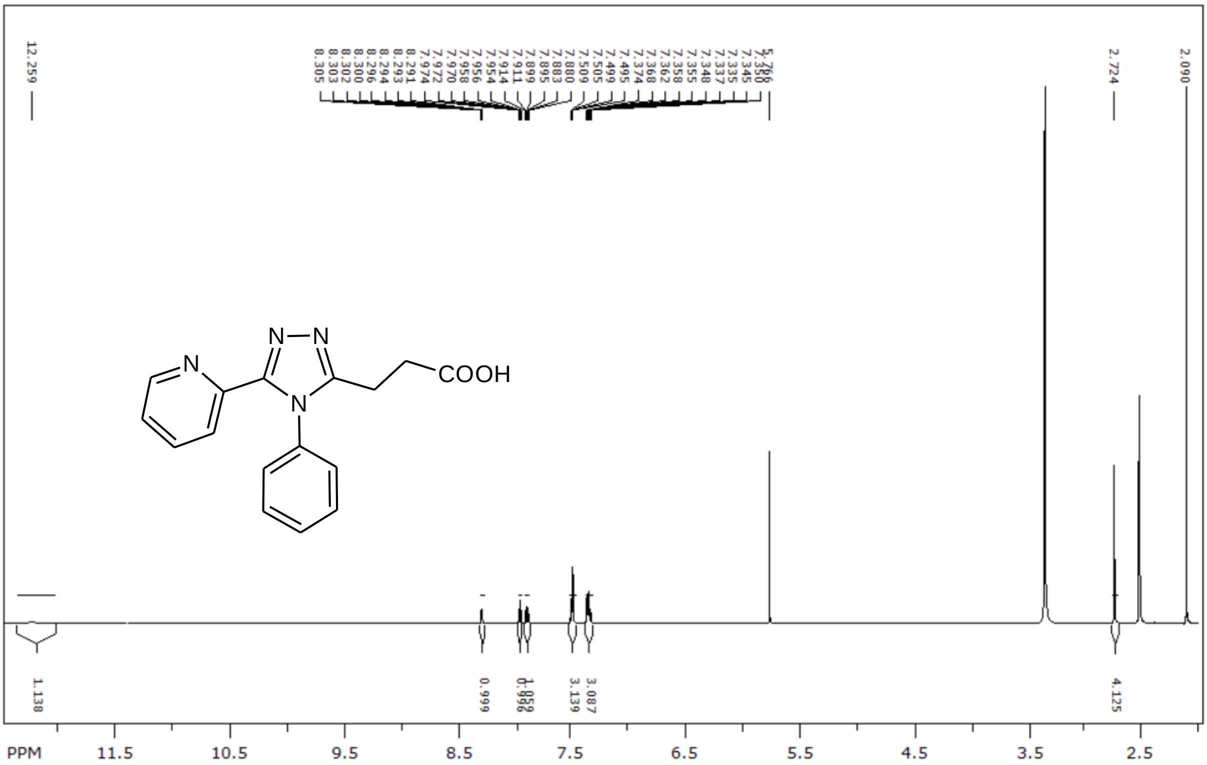


Figure S2. $^{1}$H NMR spectrum of compound **3b** (in DMSO-$d_6$)

(5.762 ppm – impurity of dichloromethane formed in the NMR study, 2.091 – impurity of acetone in the NMR study)

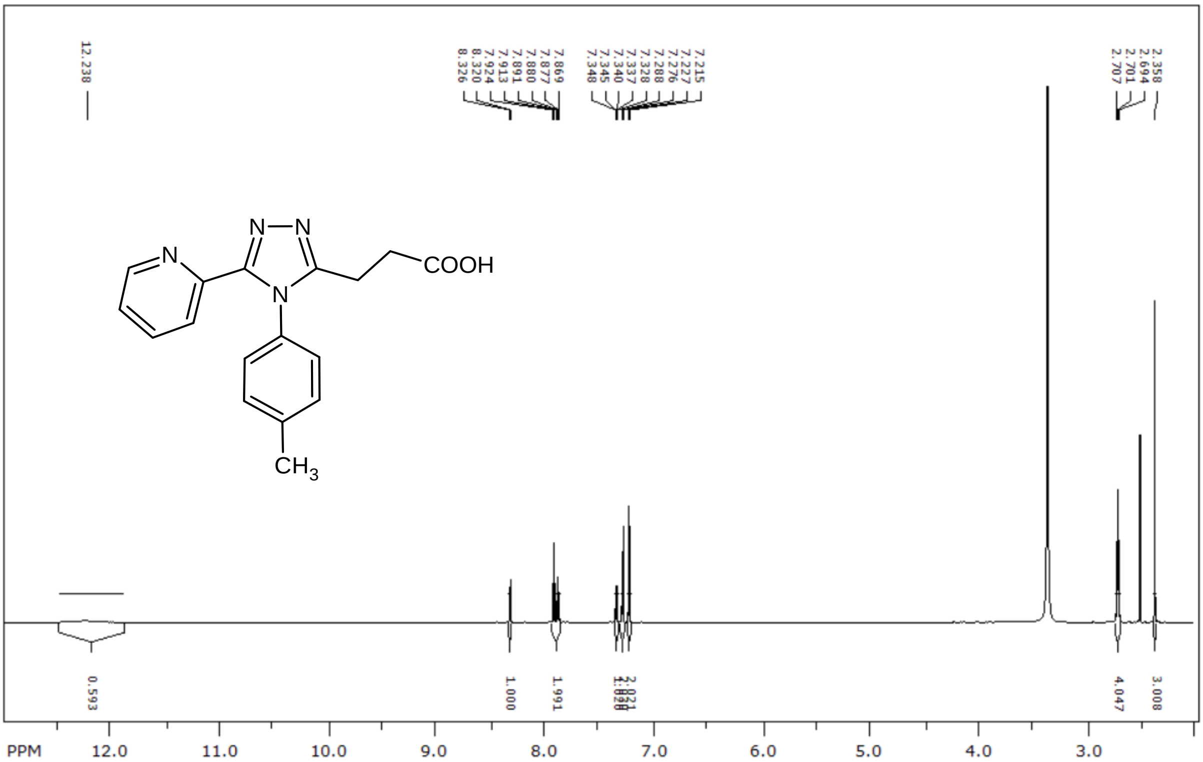


Figure S3. $^{1}$H NMR spectrum of compound **3c** (in DMSO-$d_6$)

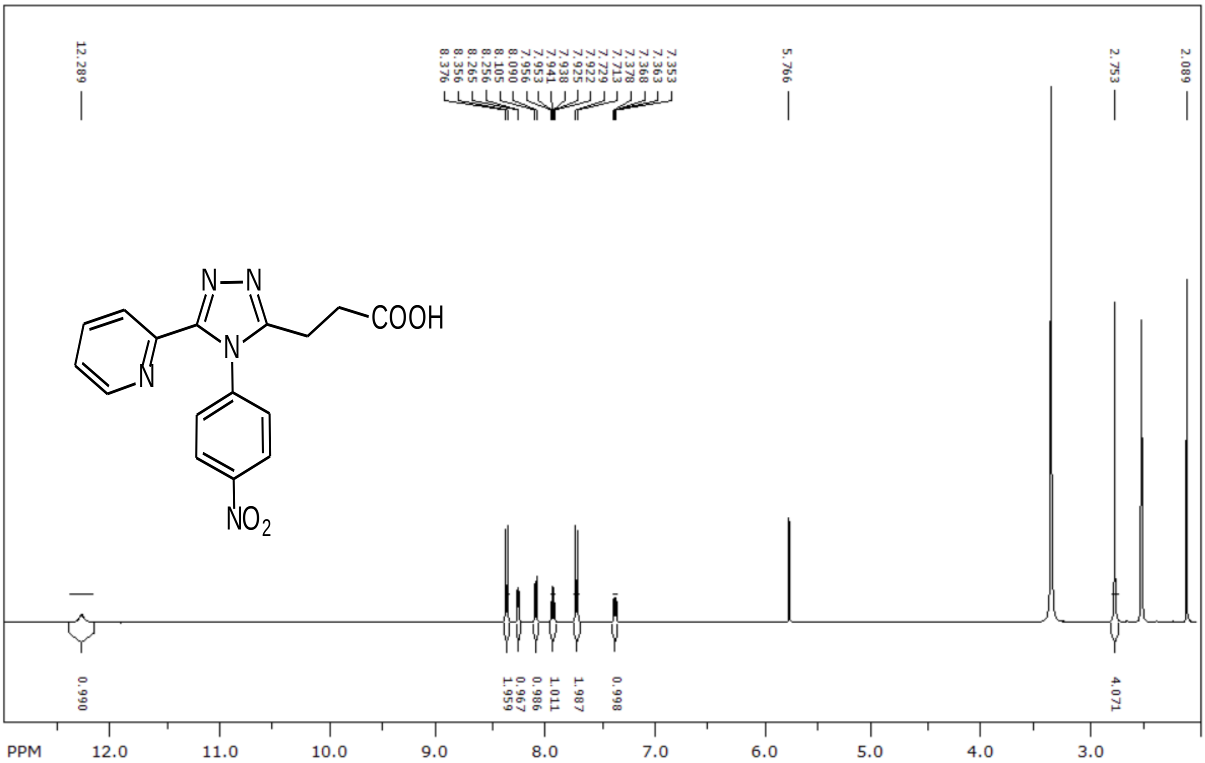


Figure S4. $^{1}$H NMR spectrum of compound **3d** (in DMSO-$d_6$)

(5.762 ppm – impurity of dichloromethane formed in the NMR study, 2.089 – impurity of acetone in the NMR study)

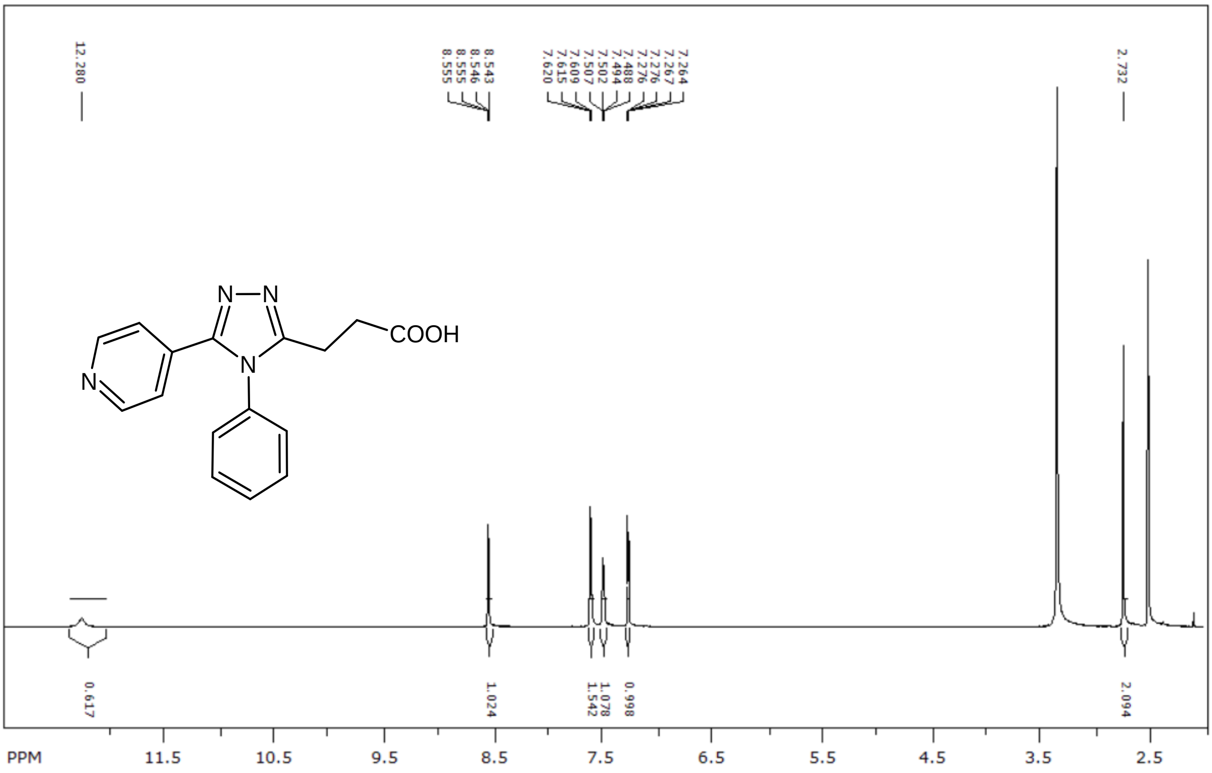


Figure S5. $^{1}$H NMR spectrum of compound **3e** (in DMSO-$d_6$)

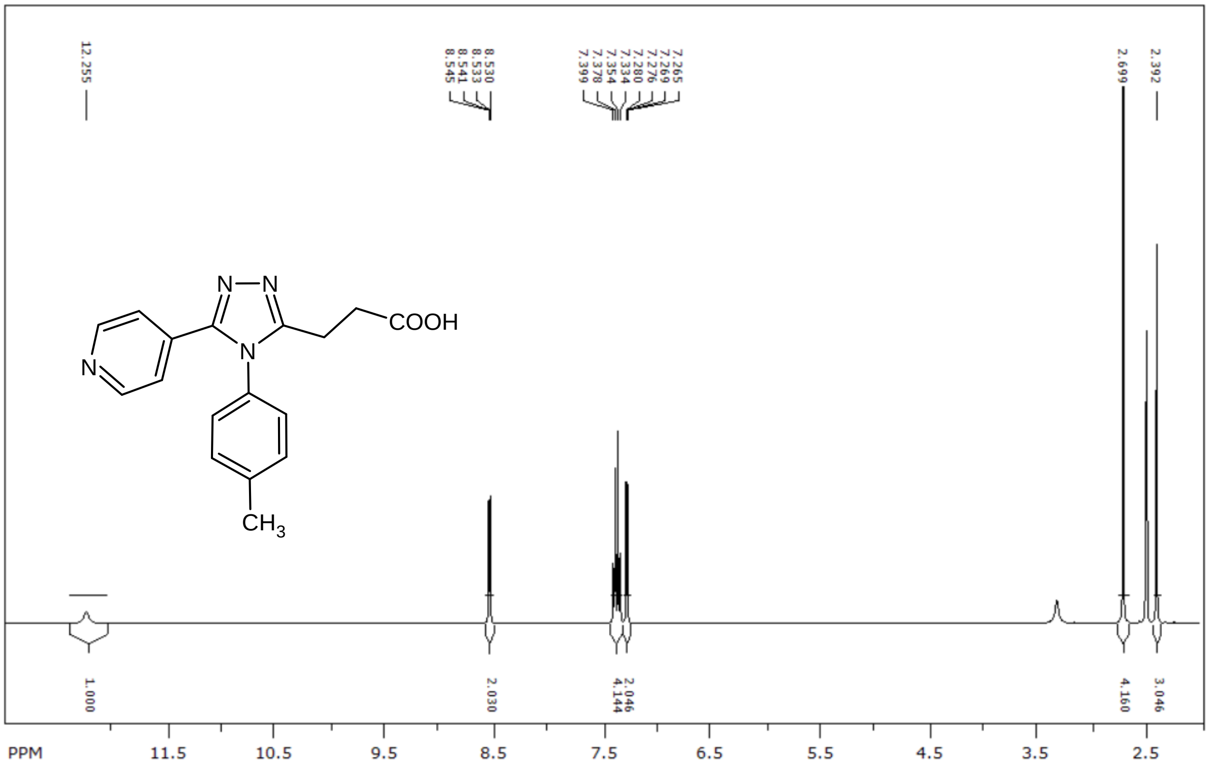


Figure S6. $^{1}$H NMR spectrum of compound **3f** (in DMSO-$d_6$)

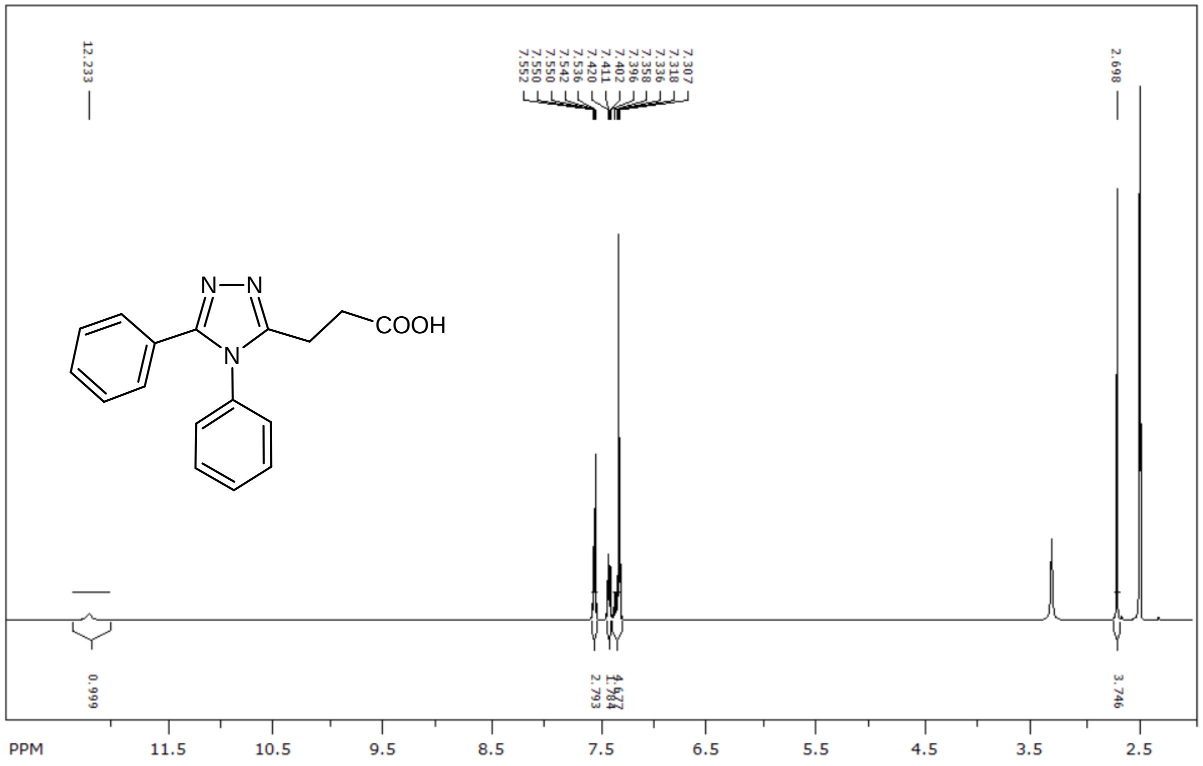


Figure S7. $^{1}$H NMR spectrum of compound **3g** (in DMSO-$d_6$)

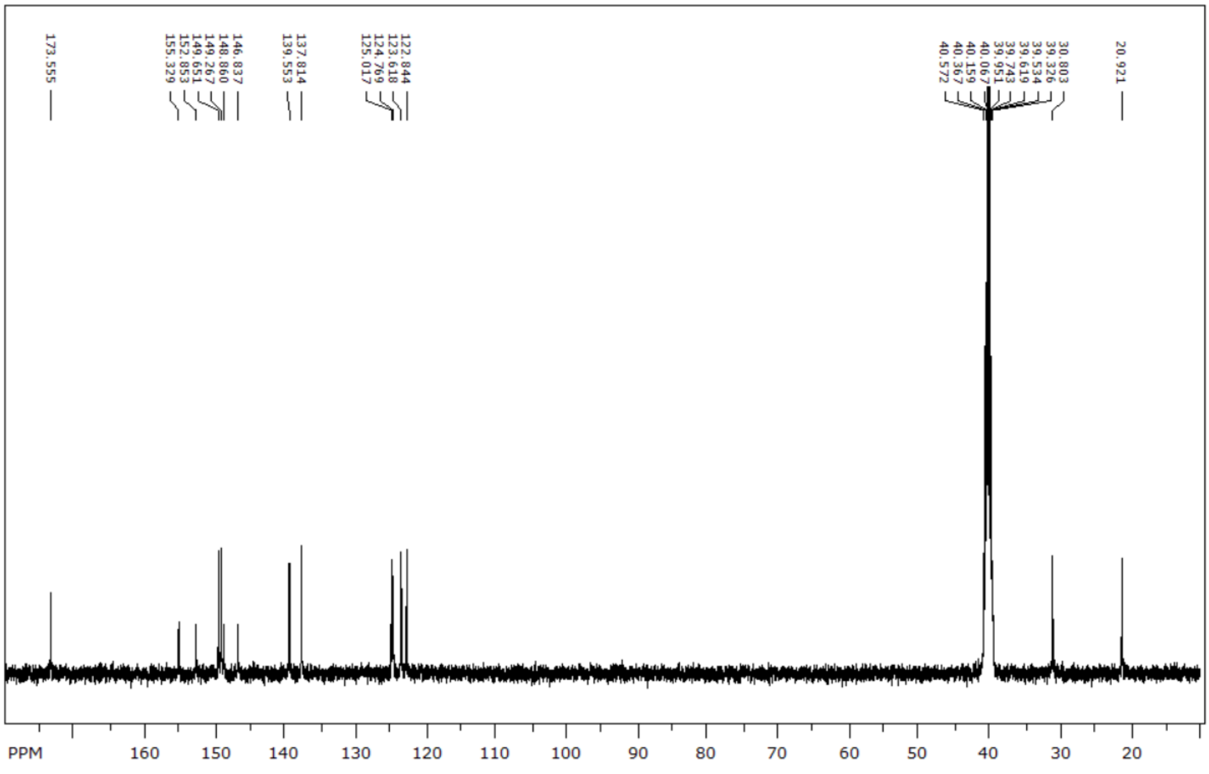


Figure S8. $^{13}$C NMR spectrum of compound **3a** (in DMSO-$d_6$)

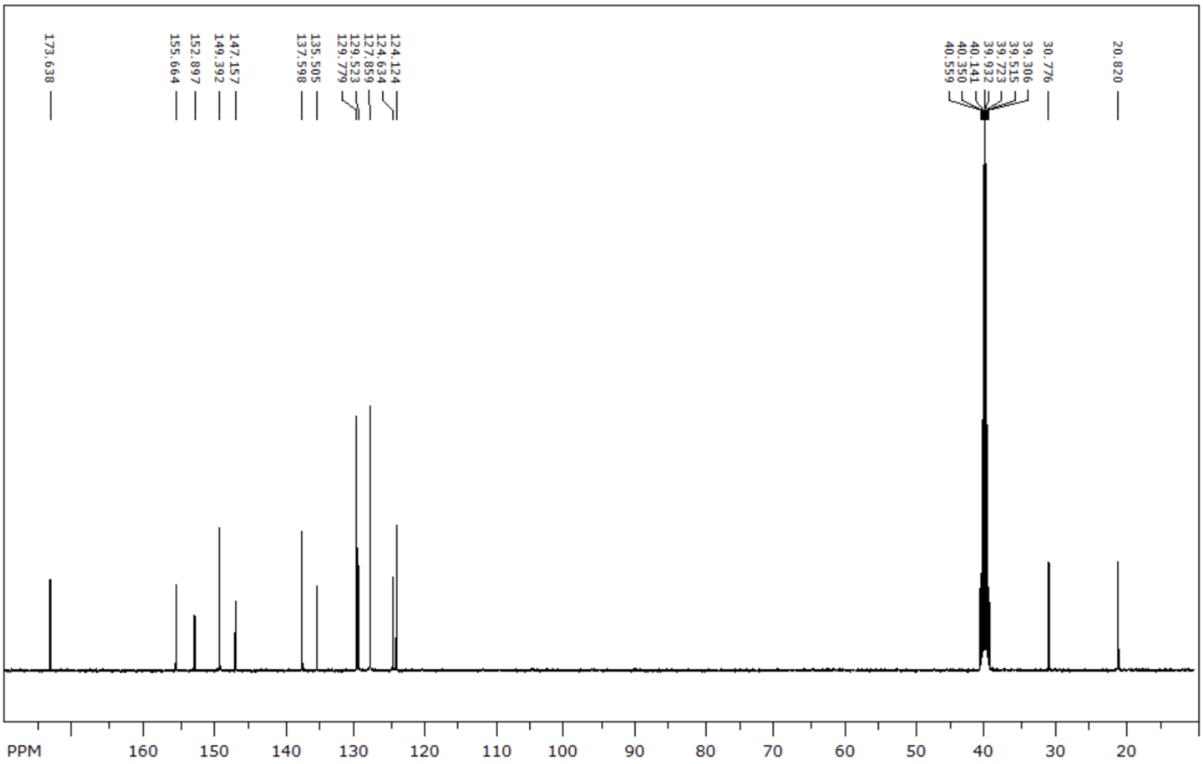


Figure S9. $^{13}$C NMR spectrum of compound **3b** (in DMSO-$d_6$)

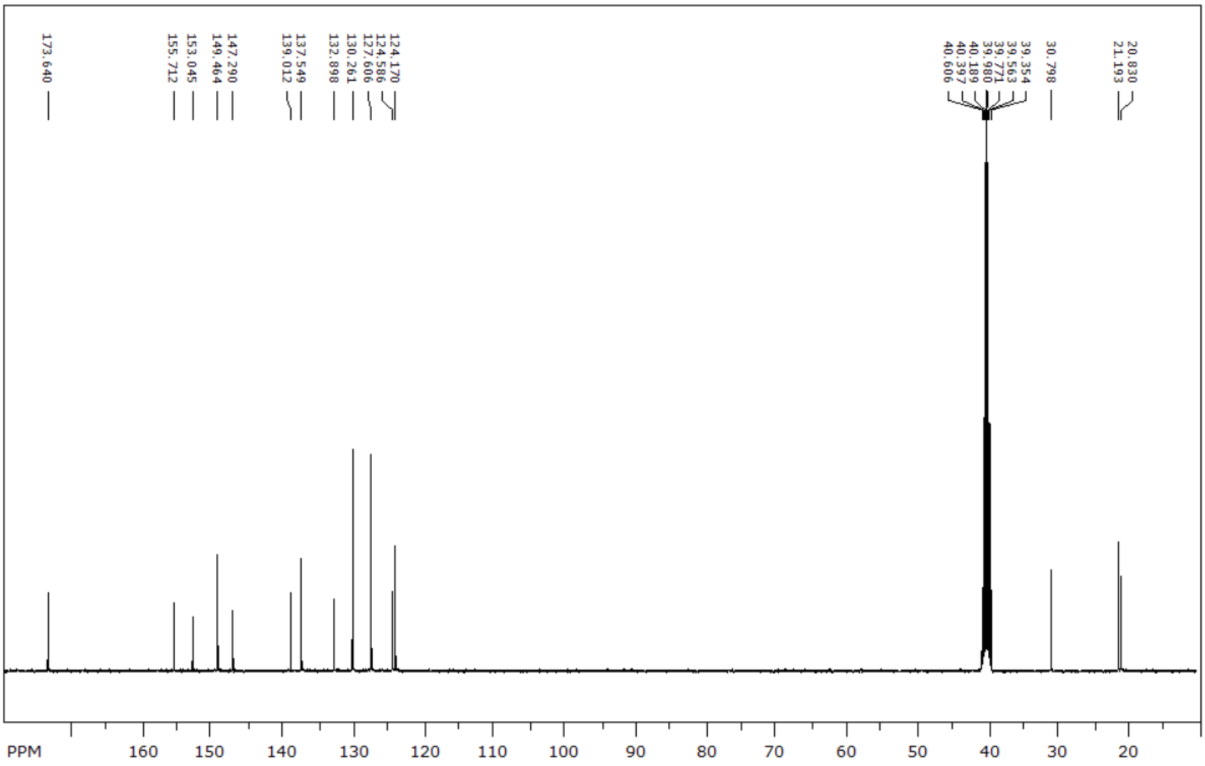


Figure S10. $^{13}$C NMR spectrum of compound **3c** (in DMSO-$d_6$)

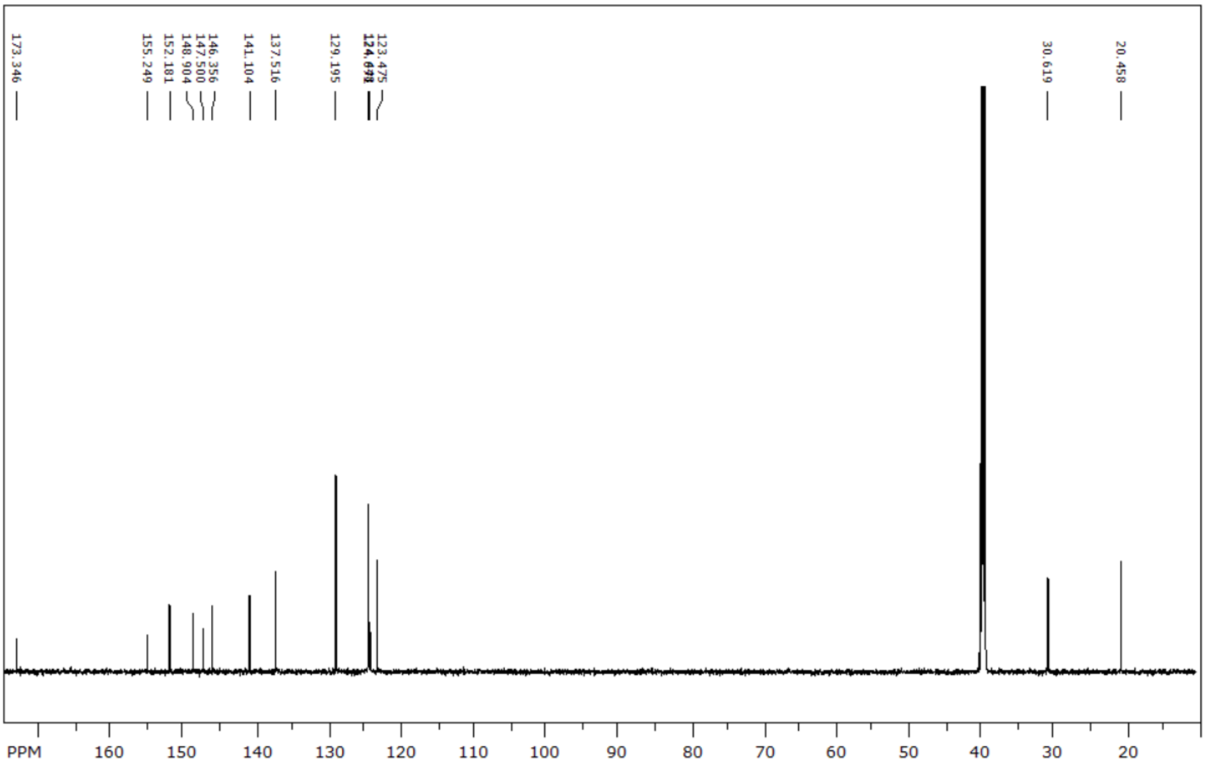


Figure S11. $^{13}C$ NMR spectrum of compound **3d** (in DMSO-$d_6$)

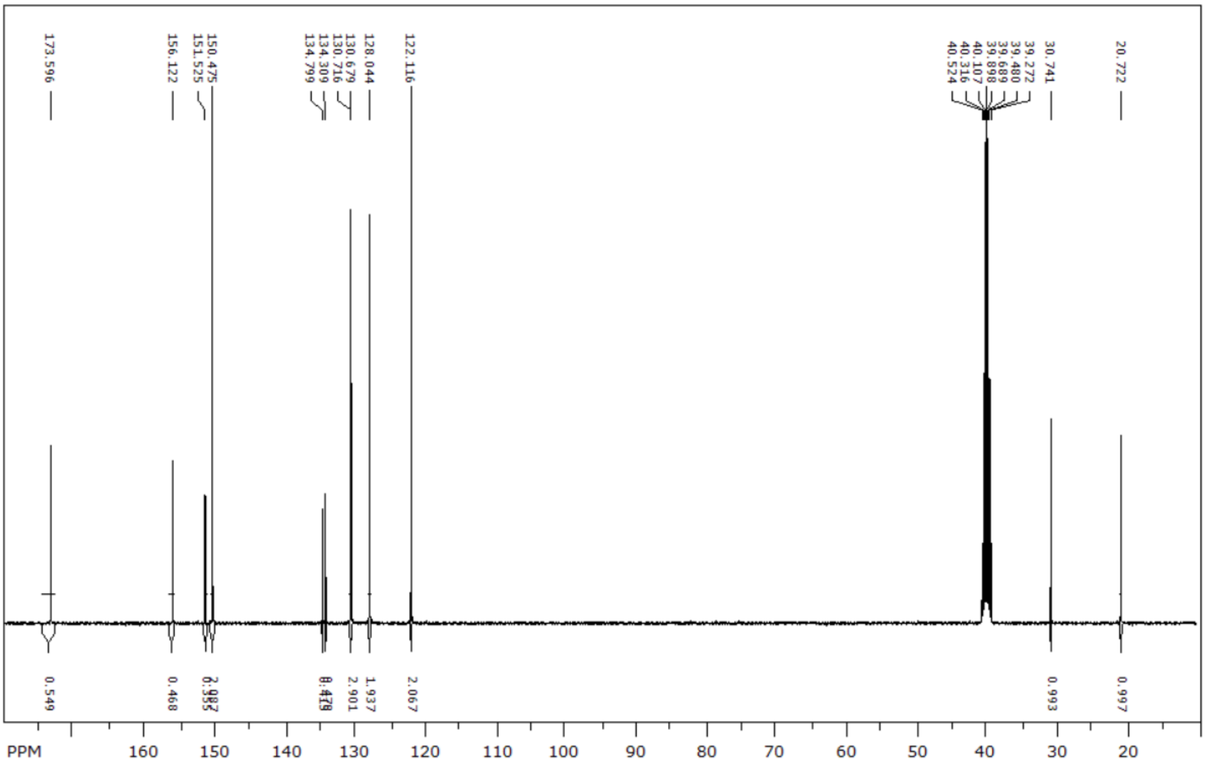


Figure S12. $^{13}C$ NMR spectrum of compound **3e** (in DMSO-$d_6$)

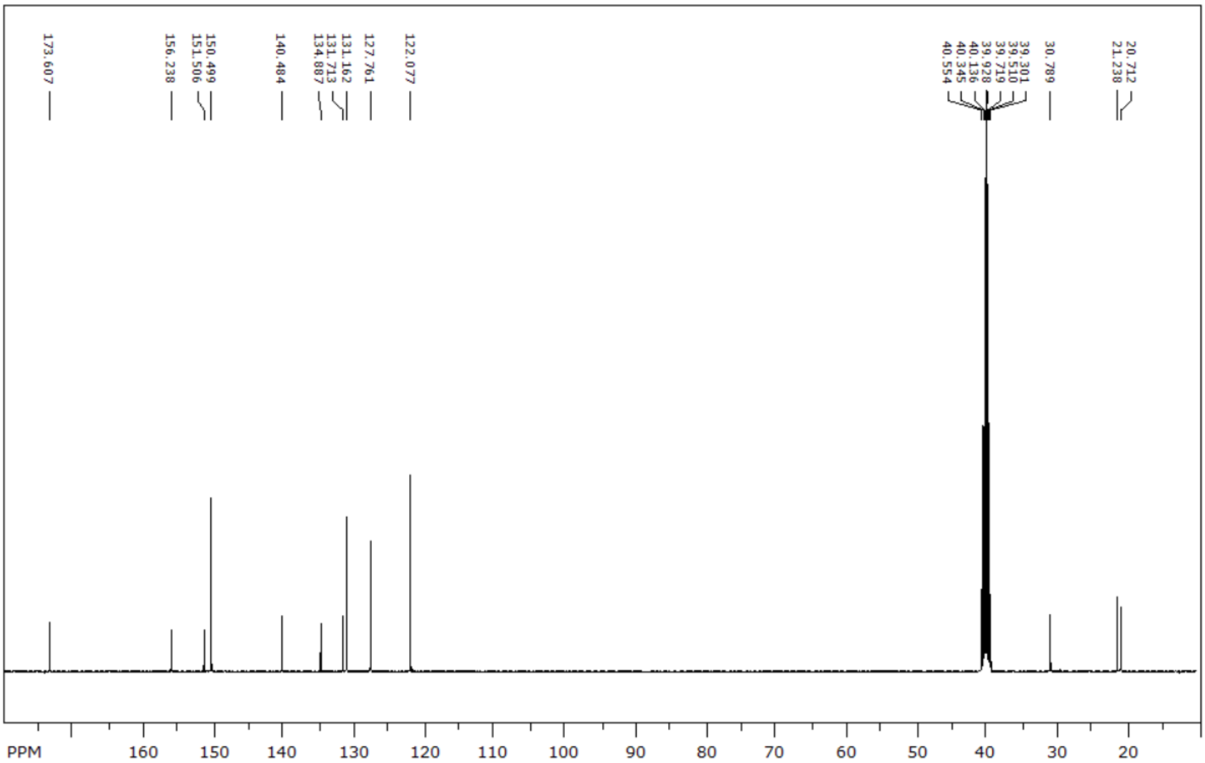


Figure S13. $^{13}$C NMR spectrum of compound **3f** (in DMSO-$d_6$)

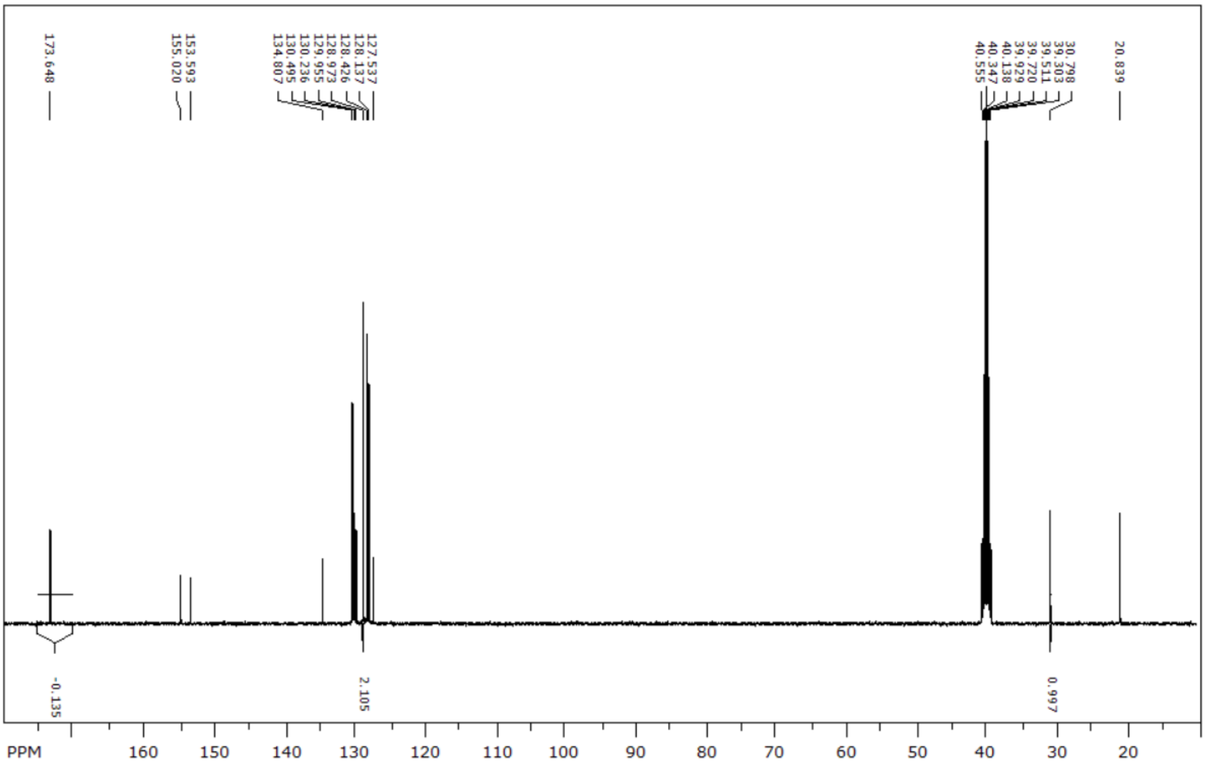


Figure S14. $^{13}$C NMR spectrum of compound **3g** (in DMSO-$d_6$)

**Elemental Composition Report** **Page 1**

**Single Mass Analysis**
Tolerance = 7.0 PPM / DBE: min = -1.5, max = 90.0
Element prediction: Off
Number of isotope peaks used for i-FIT = 9

Monoisotopic Mass, Even Electron Ions
70 formula(e) evaluated with 1 results within limits (all results (up to 1000) for each mass)
Elements Used:
C: 0-20 H: 0-25 N: 1-6 O: 0-5

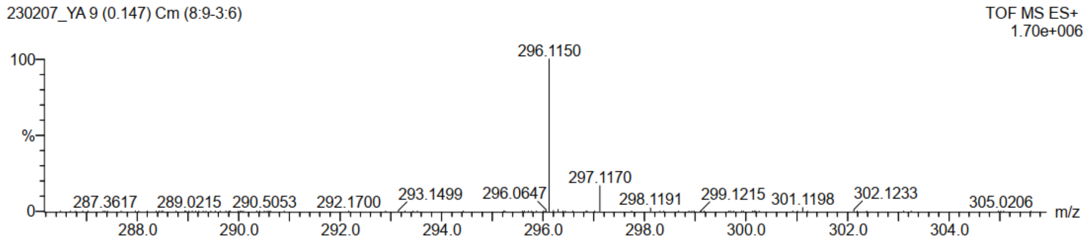


| | | | | | | | | |
|---|---|---|---|---|---|---|---|---|
| Minimum: | | | | -1.5 | | | | |
| Maximum: | | 5.0 | 7.0 | 90.0 | | | | |
| Mass | Calc. Mass | mDa | PPM | DBE | i-FIT | Norm | Conf(%) | Formula |
| 296.1150 | 296.1147 | 0.3 | 1.0 | 11.5 | 345.7 | n/a | n/a | C15 H14 N5 O2 |

Figure S15. HRMS spectrum of compound **3a**

**Elemental Composition Report** **Page 1**

**Single Mass Analysis**
Tolerance = 7.0 PPM / DBE: min = -1.5, max = 90.0
Element prediction: Off
Number of isotope peaks used for i-FIT = 9

Monoisotopic Mass, Even Electron Ions
48 formula(e) evaluated with 1 results within limits (all results (up to 1000) for each mass)
Elements Used:
C: 0-20 H: 0-25 N: 1-4 O: 0-5

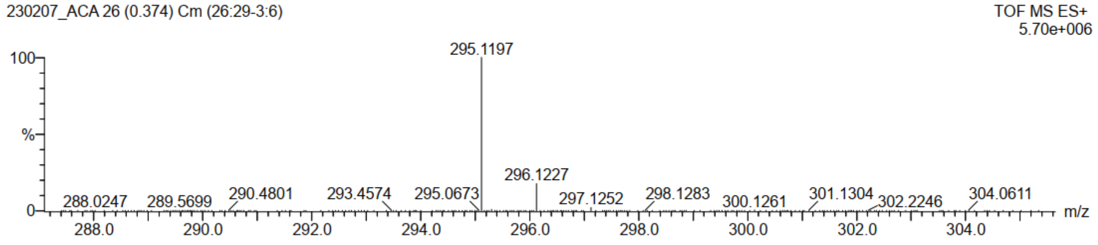


| | | | | | | | | |
|---|---|---|---|---|---|---|---|---|
| Minimum: | | | | -1.5 | | | | |
| Maximum: | | 5.0 | 7.0 | 90.0 | | | | |
| Mass | Calc. Mass | mDa | PPM | DBE | i-FIT | Norm | Conf(%) | Formula |
| 295.1197 | 295.1195 | 0.2 | 0.7 | 11.5 | 1420.2 | n/a | n/a | C16 H15 N4 O2 |

Figure S16. HRMS spectrum of compound **3b**

## Single Mass Analysis

Tolerance = 7.0 PPM / DBE: min = -1.5, max = 90.0
Element prediction: Off
Number of isotope peaks used for i-FIT = 9

Monoisotopic Mass, Even Electron Ions
43 formula(e) evaluated with 1 results within limits (all results (up to 1000) for each mass)
Elements Used:
C: 0-20 H: 0-25 N: 1-4 O: 0-5

230207_XA 40 (0.548) Cm (40:41)

TOF MS ES+
3.15e+006

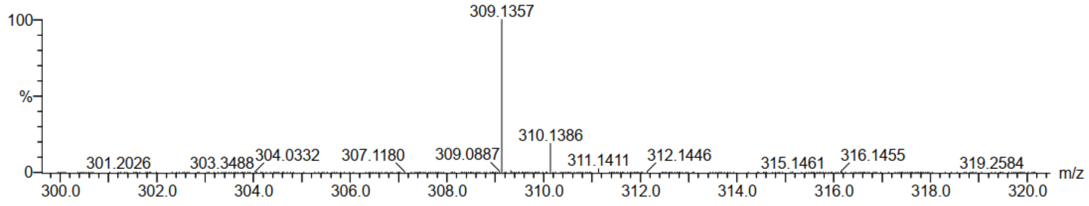


| | | | | | | | | |
|---|---|---|---|---|---|---|---|---|
| Minimum: | | | | -1.5 | | | | |
| Maximum: | | 5.0 | 7.0 | 90.0 | | | | |

| Mass | Calc. Mass | mDa | PPM | DBE | i-FIT | Norm | Conf(%) | Formula |
|---|---|---|---|---|---|---|---|---|
| 309.1357 | 309.1352 | 0.5 | 1.6 | 11.5 | 1340.5 | n/a | n/a | C17 H17 N4 O2 |

Figure S17. HRMS spectrum of compound **3c**

## Single Mass Analysis

Tolerance = 5.0 PPM / DBE: min = -1.5, max = 90.0
Element prediction: Off
Number of isotope peaks used for i-FIT = 9

Monoisotopic Mass, Even Electron Ions
185 formula(e) evaluated with 1 results within limits (all results (up to 1000) for each mass)
Elements Used:
C: 0-45 H: 0-45 N: 0-6 O: 0-6

230310_probka_AA_pos_A 25 (0.365) Cm (25:30-4:6)

TOF MS ES+
1.15e+006

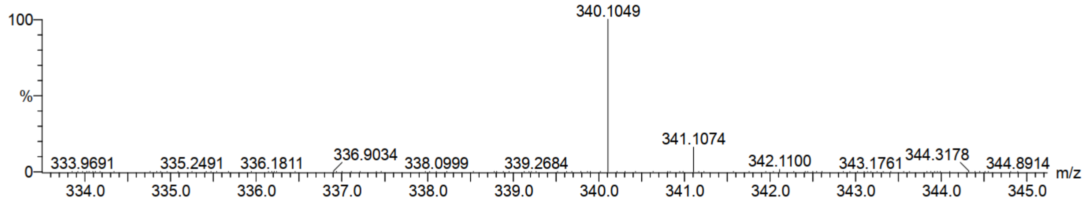


| | | | | | | | | |
|---|---|---|---|---|---|---|---|---|
| Minimum: | | | | -1.5 | | | | |
| Maximum: | | 5.0 | 5.0 | 90.0 | | | | |

| Mass | Calc. Mass | mDa | PPM | DBE | i-FIT | Norm | Conf(%) | Formula |
|---|---|---|---|---|---|---|---|---|
| 340.1049 | 340.1046 | 0.3 | 0.9 | 12.5 | 480.0 | n/a | n/a | C16 H14 N5 O4 |

Figure S18. HRMS spectrum of compound **3d**

## Elemental Composition Report



### Single Mass Analysis

Tolerance = 7.0 PPM / DBE: min = -1.5, max = 90.0
Element prediction: Off
Number of isotope peaks used for i-FIT = 9

Monoisotopic Mass, Even Electron Ions
48 formula(e) evaluated with 1 results within limits (all results (up to 1000) for each mass)
Elements Used:
C: 0-20 H: 0-25 N: 1-4 O: 0-5
230207_AEA 22 (0.313) Cm (22:27-3:6)

TOF MS ES+
2.65e+006

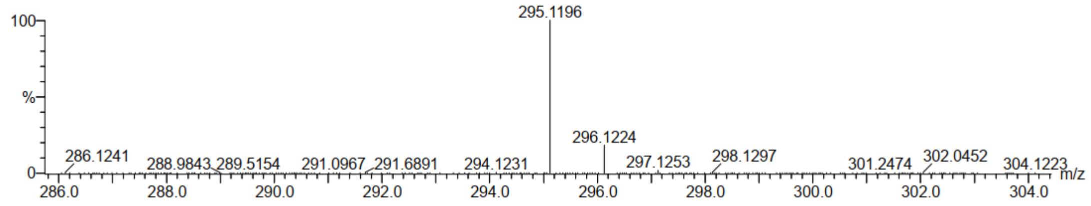


| | | | | | | | | |
|---|---|---|---|---|---|---|---|---|
| Minimum: | | | | -1.5 | | | | |
| Maximum: | | 5.0 | 7.0 | 90.0 | | | | |

| Mass | Calc. Mass | mDa | PPM | DBE | i-FIT | Norm | Conf(%) | Formula |
|---|---|---|---|---|---|---|---|---|
| 295.1196 | 295.1195 | 0.1 | 0.3 | 11.5 | 1163.8 | n/a | n/a | C16 H15 N4 O2 |

Figure S19. HRMS spectrum of compound **3e**

## Elemental Composition Report



### Single Mass Analysis

Tolerance = 7.0 PPM / DBE: min = -1.5, max = 90.0
Element prediction: Off
Number of isotope peaks used for i-FIT = 9

Monoisotopic Mass, Even Electron Ions
43 formula(e) evaluated with 1 results within limits (all results (up to 1000) for each mass)
Elements Used:
C: 0-20 H: 0-25 N: 1-4 O: 0-5
230207_ABA 23 (0.321) Cm (23:24-3:6)

TOF MS ES+
2.28e+006

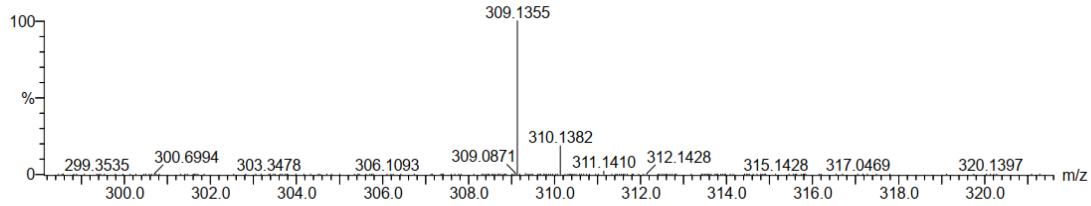


| | | | | | | | | |
|---|---|---|---|---|---|---|---|---|
| Minimum: | | | | -1.5 | | | | |
| Maximum: | | 5.0 | 7.0 | 90.0 | | | | |

| Mass | Calc. Mass | mDa | PPM | DBE | i-FIT | Norm | Conf(%) | Formula |
|---|---|---|---|---|---|---|---|---|
| 309.1355 | 309.1352 | 0.3 | 1.0 | 11.5 | 830.5 | n/a | n/a | C17 H17 N4 O2 |

Figure S20. HRMS spectrum of compound **3f**

**Elemental Composition Report** **Page 1**

**Single Mass Analysis**
Tolerance = 7.0 PPM / DBE: min = -1.5, max = 90.0
Element prediction: Off
Number of isotope peaks used for i-FIT = 9

Monoisotopic Mass, Even Electron Ions
45 formula(e) evaluated with 1 results within limits (all results (up to 1000) for each mass)
Elements Used:
C: 0-20 H: 0-25 N: 1-4 O: 0-5
230207_ADA 23 (0.321) Cm (23:24-3:6)

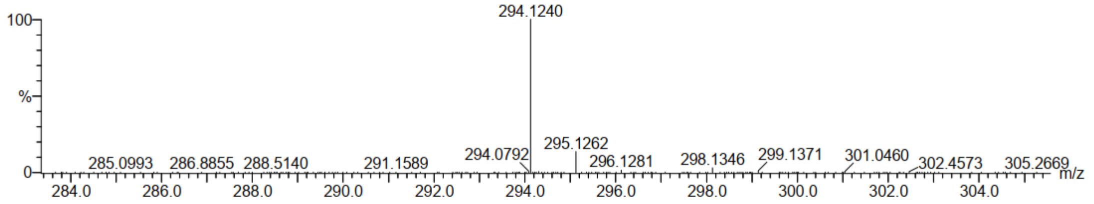


| | Mass | Calc. Mass | mDa | PPM | DBE | i-FIT | Norm | Conf(%) | Formula |
|---|---|---|---|---|---|---|---|---|---|
| Minimum: | | | | | -1.5 | | | | |
| Maximum: | | | 5.0 | 7.0 | 90.0 | | | | |
| | 294.1240 | 294.1243 | -0.3 | -1.0 | 11.5 | 849.5 | n/a | n/a | C17 H16 N3 O2 |

Figure S21. HRMS spectrum of compound **3g**

Table S2. The results of cell apoptosis assay of compounds **3a-3g** and ibuprofen at concentration 100 µg/mL in PBMC 24 h cultured measured by flow cytometry.

| | **viable cells** | **early apoptotic cells** | **late apoptotic cells** | **necrotic cells** |
|---|---|---|---|---|
| control | 96.45% | 0.58% | 0.40% | 2.56% |
| control with 2% DMSO | 94.19% | 2.08% | 0.74% | 2.98% |
| **IBU** | 96.01% | 1.39% | 0.77% | 1.84% |
| **3c** | 96.72% | 1.22% | 0.76% | 1.31% |
| **3b** | 94.89% | 1.64% | 1.51% | 1.96% |
| **3c** | 95.71% | 1.68% | 1.26% | 1.36% |
| **3d** | 94.71% | 1.84% | 1.82% | 1.62% |
| **3e** | 95.74% | 0.73% | 0.47% | 3.05% |
| **3f** | 95.34% | 1.57% | 0.73% | 2.36% |
| **3g** | 95.62% | 1.92% | 0.63% | 1.84% |

Table S3. Minimal inhibitory concentrations of compounds **3a-3g**, ciprofloxacin (CIP) and amphotericin B (AmB).

| **strain** | **MIC µg/mL** | | | | | | | | |
|---|---|---|---|---|---|---|---|---|---|
| | **3a** | **3b** | **3c** | **3d** | **3e** | **3f** | **3g** | **CIP** | **AmB** |
| *E. coli* ATCC 25922 | >512 | 512 | >512 | 512 | >512 | 512 | 256 | 0.015 | |
| *Y. enterocolitica* O3 | 512 | 512 | 512 | 512 | 512 | 512 | 512 | 0.125 | |
| *S. aureus* ATCC 25923 | >512 | >512 | >512 | >512 | >512 | >512 | 256 | 0.25 | |
| *M. smegmatis* | 128 | 256 | 256 | 512 | 128 | 256 | 512 | 0.5 | |
| *C. albicans* ATCC 90028 | >512 | >512 | >512 | 512 | >512 | 512 | 512 | - | 0.125 |
